# Preparation and characterization of antibacterial fibrous membranes composites based on green synthesized nanoparticles loaded on electrospun polyacrylonitrile fibrous membranes

**DOI:** 10.1038/s41598-026-51833-z

**Published:** 2026-05-19

**Authors:** Thanaa Ibrahim Shalaby, Ola Mahmoud, Asmaa Abd El kader

**Affiliations:** 1 Department of Medical Biophysics, Medical Research Institute, Alexandria University, Alexandria, Egypt; 2 Nanotechnology Training Center, Medical Technology Center, Alexandria University, Alexandria, Egypt; 3 Microbiology Department, Medical Research Institute, Alexandria University, Alexandria, Egypt; 4 Basic Science Department, Faculty of Physical Therapy, Pharos University in Alexandria, Alexandria, Egypt

**Keywords:** Electrospinning, Nanofibers, Green synthesis, Antibacterial activity, mechanical properties, disinfection, filtration, Chemistry, Materials science, Nanoscience and technology

## Abstract

The functionalization of electrospun nanofibers with a wide range of materials allows them to be used for a variety of applications. In this work, Ag NPs, ZnO NPs and CuO NPs were synthesized through green methods and characterized then incorporated separately in PAN nanofibers using electrospinning technique to fabricate antibacterial membranes with high-dispersed green synthesized nanoparticles. The nanofibers composites PAN/AgNPs, PAN/ZnO NPs and PAN/CuONPs and plain PAN were characterized using SEM, FTIR, EDX, furthermore, antibacterial properties and mechanical properties were also analyzed. The nanofiber composites were used for filtration of contaminated drinking water. The results indicated that the surface morphology of PAN loaded with NPs became uneven, rough and irregular compared with plain PAN. The mechanical properties of PAN can be effectively enhanced with the addition of NPs. The cumulative amounts of the released NPs and the bactericidal activities of the nanofiber composites against Gram negative *Escherichia coli* was evaluated. All samples exhibited antibacterial properties; however, the sample with Ag NPs exhibited better antibacterial activity. By considering the excellent results of nanofibrous composites, they can therefore be recommended for water disinfection applications.

**Supplementary Information:**

The online version contains supplementary material available at 10.1038/s41598-026-51833-z.

## Introduction

 Globally, water scarcity is one of the foremost health and environmental challenges faced. Climate change and drastically increasing population is threatening the availability of potable water, with detrimental environmental, social and economic impacts. conventional methods of water treatment are not meeting increasing water demands, thus, research into new water treatment technologies are of utmost importance. Water sanitation, reclamation and decontamination methods that are lower in cost and are more efficient than current water treatment options need to be developed and expanded to a level where it can alleviate water stress, especially in 3rd world countries, where access to potable water is often a luxury^1–3^.

Filtration is a commonly used method that removes impurities by passing water through various physical barriers such as activated carbon filters, ceramic filters, and membrane filters^4^. Ultrafiltration using semi-permeable membrane with pore size ranging from 0.1 to 0.001 micrometers effectively separate suspended solids, colloids, bacteria, viruses, and other macromolecules from water^5^, without the need for chemical additives or disinfectants, making it a reliable technology for producing safe drinking water^6^. Nanofibers have excellent filtration properties, and due to the variety of polymers that can be used to fabricate nanofibers, and the versatility of being able to add functional molecules and chemical groups to the nanofibers, make nanofibers applicable to sanitation and purification of water. Nanofibers are produced from a range of electrospinnable polymers by the process of needle-electrospinning.

Electrospinning is a straightforward method to fabricate fibers with large surface area and porosity in the nanometer range. Recently, the unique properties of electrospun fibres have attracted a great deal of attention for a broad range of applications from particle filtering in membrane science, to cell culturing in tissue engineering^7,8^. Electrospinning involves the application of an electric field to a droplet or a jet of polymeric solution or melt, causing the formation of ultrafine fibers through a process of electrostatic stretching and elongation. The resulting fibers have diameters ranging from a few nanometers to several micrometers^9^. The pore size of membranes can be controlled by the electrospinning technology. The porosity of electrospun membranes impacts properties such as permeability, adsorption capacity, and mechanical strength^10,11^. Electrospinning allows for the incorporation of various materials, additives, or functional groups into the polymer solution, thereby enabling control over the surface chemistry of the resulting membranes. Furthermore, the electrospinning process allows for texact control over fiber morphology and the incorporation of nanoparticles, enabling the development of tailored membranes with enhanced performance for water disinfection applications^12,13^. Anti-microbial nanofibers can be synthesized by incorporating nanobiocides such as silver, ZnO or CuO nanoparticles into the nanofibers. The combination of the high specific surface area and fineness of electrospun nanofibers with the biocidal activity of Ag, ZnO or CuO nanoparticles results in a superior and versatile antimicrobial nanofibers composites.

Nanofibrous composite membranes are advanced materials that consist of a combination of nanofibers and other materials (such as polymers or nanoparticles) to meet specific requirements. The resulting nanofibrous composite membranes possess several advantageous characteristics such as, their large surface area-to-volume percentage enables efficient mass transport and separation processes. The small pore size and tortuous structure of the nanofibers provide excellent filtration performance, allowing the membranes to effectively remove particles, bacteria, viruses, or other contaminants from contaminated water. Such nanofibrous composite membranes are highly resistant to fouling, which refers to the accumulation of contaminants on the membrane surface affecting filtration process^14^.

The presence of metal oxide nanoparticles in the membrane structure can improve the filtration efficiency^7^. Metal oxide nanoparticles can possess self-cleaning properties which helps to reduce fouling and prolong the lifespan of the membrane by inhibiting microbial adhesion and growth. Metal oxide nanoparticles can provide durable and long-lasting antimicrobial effects even after repeated use and exposure to water, ensuring continuous protection against microbial contamination^15,16^. The antibacterial activity of zinc oxide, copper oxide, and silver nanoparticles can be leveraged to improve the membrane’s ability to kill or inhibit the growth of microorganisms. These membranes can be utilized in various water treatment processes, including filtration, ultrafiltration, and membrane distillation, to improve the disinfection efficiency and ensure the production of safe and clean water^17,18^.

Polyacrylonitrile (PAN) possesses high mechanical strength thanks to the strong hydrogen bonding of its molecular structure. It also exhibits strong chemical resistance, excellent acid-proof alkalinity, good thermal stability, resistance to sunlight, a diminished susceptibility to humidity, and low cost. It is widely used in the preparation of microfiltration (MF), ultrafiltration (UF) and hollow fiber membranes. Several chemical surface modification reactions, such as amination, reduction, hydrolysis, and amidoximation have been carried out on polyacrylonitrile nanofibers to expand their application in healthcare and hygiene products^19^.

Our previous work in this field was performed using PAN nanofibers membraned loaded with chemically prepared Ag NPs, ZnO NPs and CuO NPs^20^. In the present work all of additive nanoparticles were green synthetized; ZnO NPs and CuO NPs were synthetized using plant extract (sumac extract). To the best of our knowledge, no study has been published yet on the preparation of CuO NPs using sumac fruit extract, and Ag NPs was synthetized by photoreduction method. Green nanoparticle synthesis represents a straightforward, eco-friendly, and cost-effective method using nontoxic and environmentally benign compounds and can minimized the cytotoxicity of NPs these NPs have well-defined size and morphology^21^. Plant extracts contain various biomolecules, such as flavonoids, terpenoids, alkaloids, and polysaccharides, act as reducing and stabilizing agents during nanoparticle synthesis^22^.

The aim of this study is to prepare antimicrobial PAN nanofibers containing green synthesized Ag, ZnO or CuO nanoparticles by simple electrospinning technique, for efficient and potent water disinfection. *E. coli* was chosen as indicators of fecal contamination. The filtration performance of the fabricated hybrid membrane was tested using designed prototype filtration unit.

## Results and discussion

Water is an essential need for all living organisms on the earth; thus, its contamination has a deleterious effect on life generally. Unfortunately, only 2% of the total water sources on the planet can be safely used for drinking purposes^23^.

Membrane-based contaminant removal techniques such as nanofiltration have become increasingly popular worldwide for water purification. Electrospinning is probably the most attractive technology for fabrication of nanofibers due to its easiness and low cost^24^.

In the present study, we used PAN polymer to fabricate nanofibers membranes fabricated by electrospinning and loaded with green synthesized ZnO, CuO and Ag NPs to separate and kill bacteria from contaminated drinking water.

### Preparation of Ag NPs by photoreduction

Preparation of nanoparticles by photoreduction methods is feasible and cost effective method. Photo- reduction methods have the following advantages: (i) controlled reduction of metal ions can be carried out without using excess of reducing agent; (ii) radiation is absorbed regardless of the presence of light absorbing solutes and products and (iii) photo assisted methods are more competitive and cost effective. Another advantage of the photo induced reduction method lies on the high purity and particle size of the nanoparticles that can be controlled by wavelength and irradiation intensity without being supported by the expensive equipment^25^.

The reduction of Ag^+^ ions into elemental Ag^o^ takes place through exposure of AgNO_3_ to UV irradiation (wavelength 254 nm and power 20 W). The photoreduction of Ag^+^ ions could proceed according to the following mechanism:

1 $$AgNO3 - - - - - - - - - - - - - - - - - - A{g^o}+NO2+{1/2}\: O_2$$

AgNO3 is photosensitive and decomposes in the presence of light, the photoreduction method is simple and inexpensive when compared with other reduction methods. The photoreduction was confirmed during preparation by observing the color change; the color of solution changed from colorless into brownish yellow. Synergistic effects of electrons and UV irradiation is a more effective and fast approach to form well-dispersed Ag nanoparticles in solutions^26^. The growth of NPs was confirmed by the subsequent analyses (TEM, UV–Vis spectroscopy and XRD).

## Biosynthesis and ZnO NPs and CuONPs

Green synthesis of NPs is environmentally friendly, facile, and devoid of expensive, harsh, and toxic chemicals^27^. The utility of plant based phytochemicals in the overall synthesis and architecture of nanoparticles and various nanoparticle embedded products is highly attractive as it brings an important symbiosis between natural/plant sciences and nanotechnology^28^. The *R. coriaria* fruit extract represents an excellent and eco-friendly choice for biosynthesizing ZnO NPs and CuO NPs. It displays a dual role by acting as a reducing agent and stabilizer, enhancing the successful synthesis of the NPs. The formation of NPs was confirmed through visual observation of the solution color change. When *R. coriaria* (sumac) fruit extract was added to the aqueous of zinc acetate dihydrate (Zn (C2H3O2)2.2H2O), the color change to yellowish gray, followed by off-white, indicating the ZnO NPs formation. On the other hand, for the first time, when *R. coriaria* fruit extract was added to the aqueous copper sulfate solution, the color changed from blue to pale yellowish-green, and on vigorous stirring, the solution changed its color to dark brownish-green. After that, the optical properties and other characteristics were extensively investigated through the subsequent analyses.

## Physicochemical characterization of NPs

### TEM analysis

**Fig. 1:**
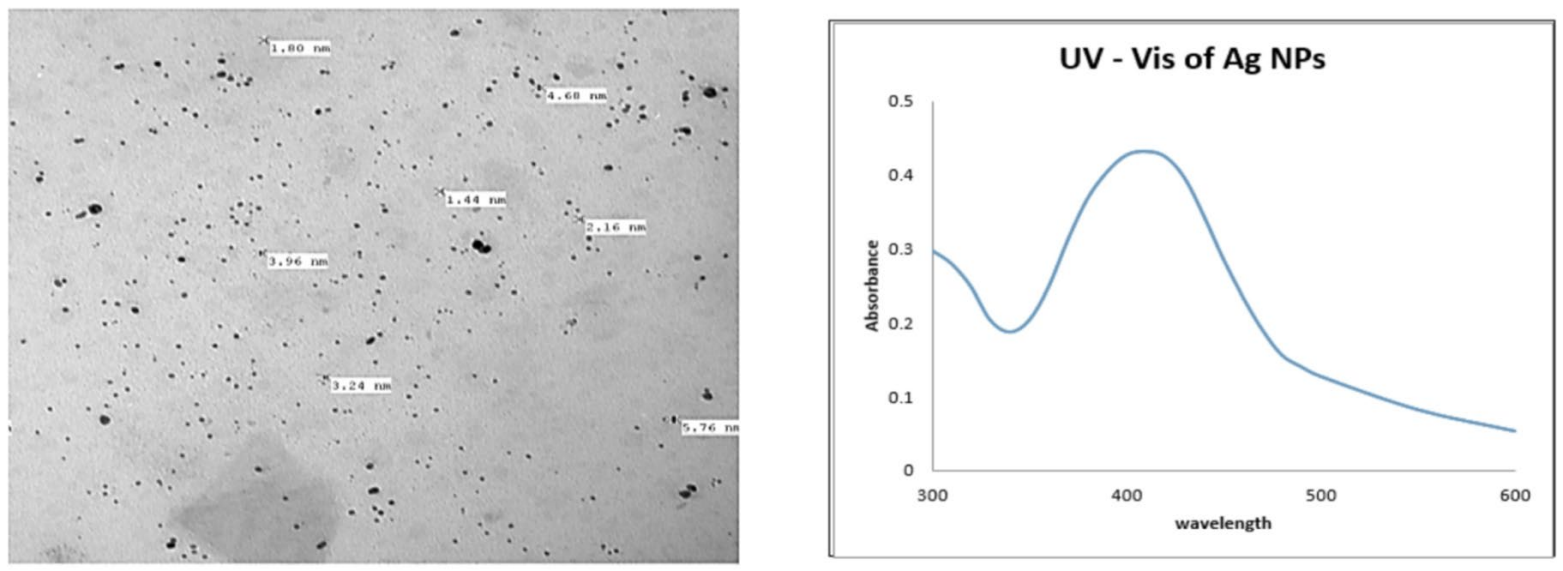
TEM images of photoreduced Ag NP and its UV-Vis absorption spectrum.

Figure (1) Illustrate the micrographs of silver nanoparticles synthesized by photo reduction of AgNO3 using UV irradiation, the as-formed Ag NPs were spherical in shape with narrow size distribution, the particle sizes ranging from 1.44 nm to 5.76 nm with an average size 3.1 nm.

The morphological nature of the *sumac fruit* extract mediated ZnO and CuO nanoparticles were analyzed using TEM. The obtained results show that the particles were agglomerated, overlapped, and distributed non-homogeneously. It may be due to the interaction of the bioactive and phytochemical compounds present in the fruit extracts.

**Fig. 2:**
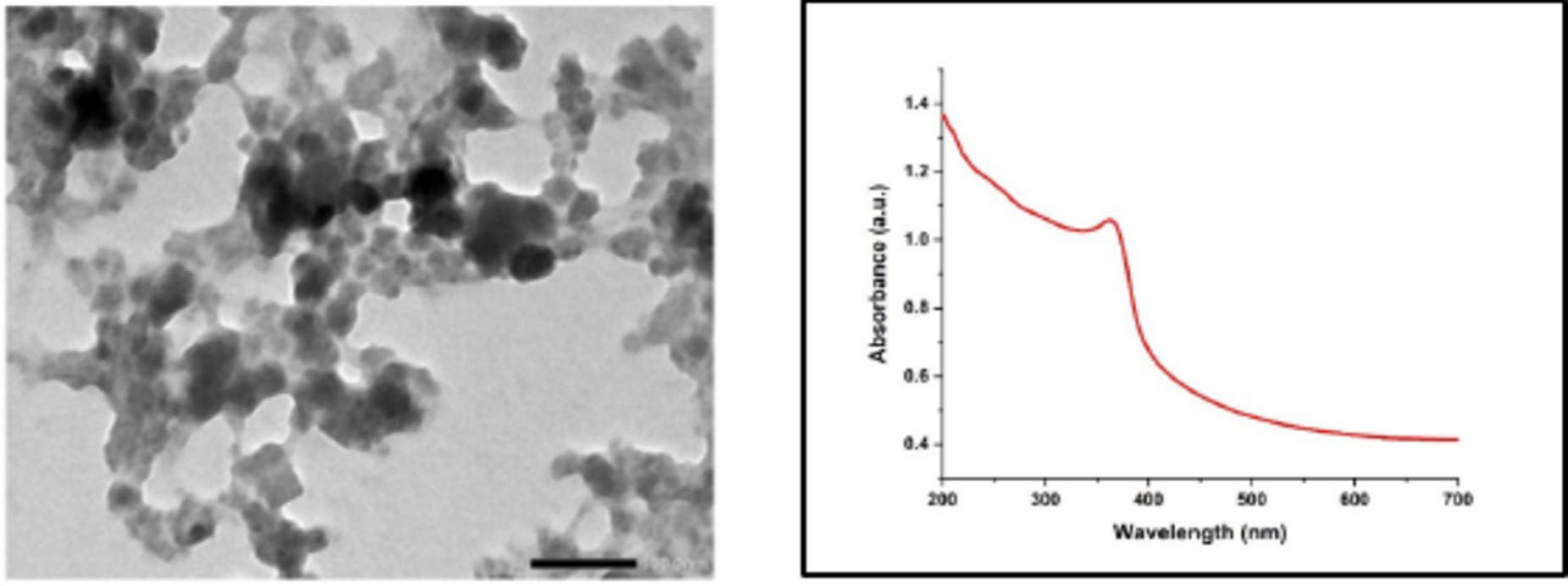
TEM of sumac mediated ZnONPs and its UV-Vis absorption spectrum.

As a result, ZnO NPs possessed spherical and hexagonal morphologies with mild aggregations as shown in Fig. 2, this result aligns with prior reports^29,30^. The micrograph displayed that the NPs aggregates are coated with a thin organic layer sourced from the fruit extract, which acts as reducing and capping organic agent. The particle size of the NPs was in the range of 12–30 nm with an average of 20.51 ± 3.90 nm.

**Fig. 3:**
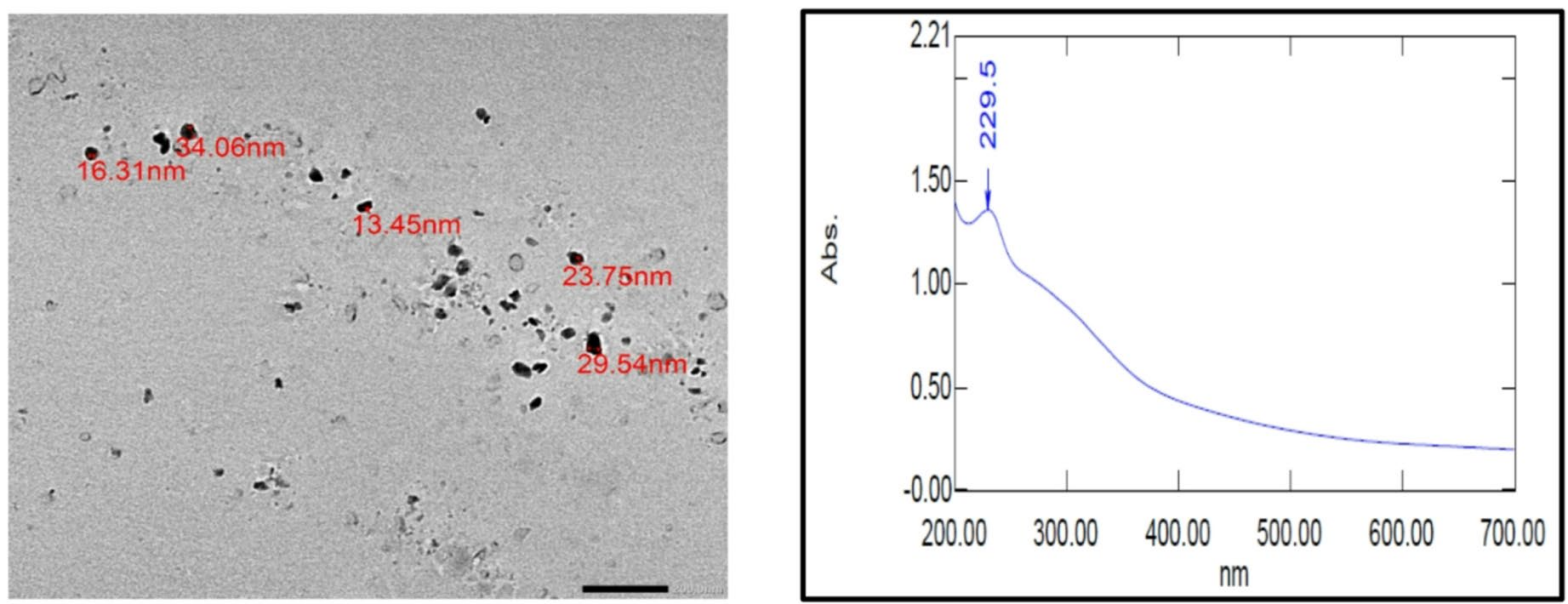
TEM of sumac mediated CuO NPs and its UV-Vis absorption spectrum.

The shape and size of sumac mediated CuO NPs were revealed by TEM analysis. The particles were in agglomerated cluster structure as depicted in the Fig. 3. The TEM image shows some particles are small spherical shapes and others are small cubical shapes with average size of 20 nm. These results are in accordance with that green synthesized CuO NPs obtained by others^31,32^.

## Optical properties

Initially, UV–Vis spectroscopy was conducted to validate the formation NPs in the wavelength region 200–600 nm at room temperature.

The UV–visible absorption spectrum of Ag NPs revealed a surface plasma peak at 430 nm (Fig. 1) due to the surface plasmon resonance band of Ag NPs. The absorption of incident light is a result of the collective oscillation of free conduction band electrons^33^.

The UV–Vis absorption spectrum of ZnO NPs revealed that the maximum absorbance existed at approximately 363 nm (Fig. 2), representing the distinctive peak absorbance value for ZnO NPs due to the surface plasmon resonance (SPR) of ZnO NPs^34^. Additionally, no additional peaks were observed in the spectrum, confirming the successful formation of pure ZnO NPs.

UV–Vis spectrum of CuO NPs synthetized using *R. coriaria* fruit extract showed a distinct peak at 229.5 nm (Fig. 3) similar to other studies on the green synthesis of CuONPs using plant extract^35,36^.

### XRD analysis

**Fig. 4:**
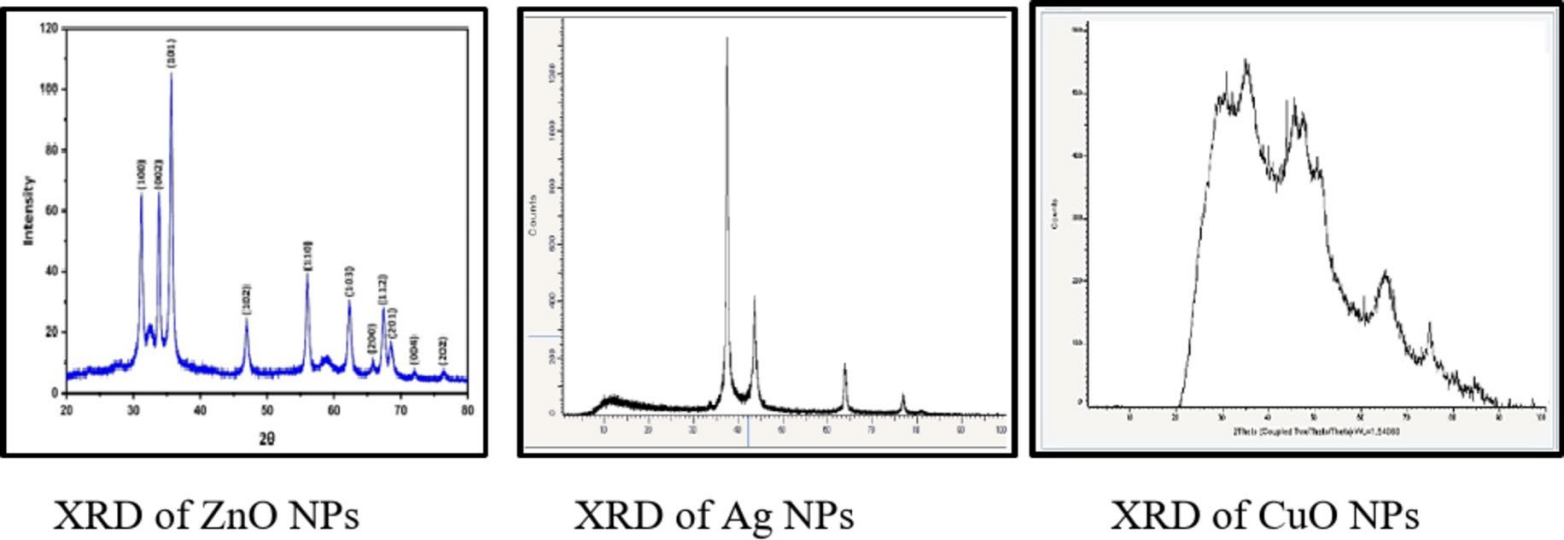
XRD of sumac mediated ZnO NPs, CuO NPs and photoreduced Ag NPs.

Crystalline nature of the prepared nanoparticles was identified from their corresponding powder XRD patterns. As depicted in Fig. 4, the XRD pattern displayed diffraction peaks of the synthesized Ag NPs by photoreduction method. The XRD spectrum showed three prominent crystalline peaks at 37^o^ (2θ) and 47^o^ (2θ) and 70^o^ (2θ), which confirms the formation of silver NPs.

Figure 4, the XRD spectrum of the green synthesized ZnO NPs using sumac extract. The diffractogram showed three intense diffraction peaks with 2θ values of 31.68°, 34.34°, and 36.17°, which are attributed to (100), (002), and (101) crystallographic reflection planes. Moreover, other strong diffraction peaks were also observed, showing 2θ values of 47.45°, 56.53°, 62.81°, 66.32°, 67.92°, 69.03°, 72.56°, and 76.93°, which correspond to (102), (110), (103), (200), (112), (201), (004), and (202) crystallographic reflection planes, respectively. These diffraction planes are in agreement with the indicated values of the hexagonal ZnO wurtzite crystalline structure (JCPDS No. 36–1451)^34^.

XRD pattern analysis revealed the crystalline nature of green synthesized CuONPs using sumac extract as shown in Fig. 4. Diffraction peaks with 2Ө values of 31.6°, 35.45°, 44.34°, 48.92°, 61.99° and 66.49° respectively, were indexed to (210), (002), (202), (020), (022) and (113) planes, respectively. The planes are in good agreement with standard diffraction data (JCPDS-80-1916) and confirming the formation of a monoclinic crystalline structure^37^.

## Characterization of nanofibers hybrid composites

### Effect of PAN polymer concentration on the microstructure of nanofibers

**Fig. 5:**
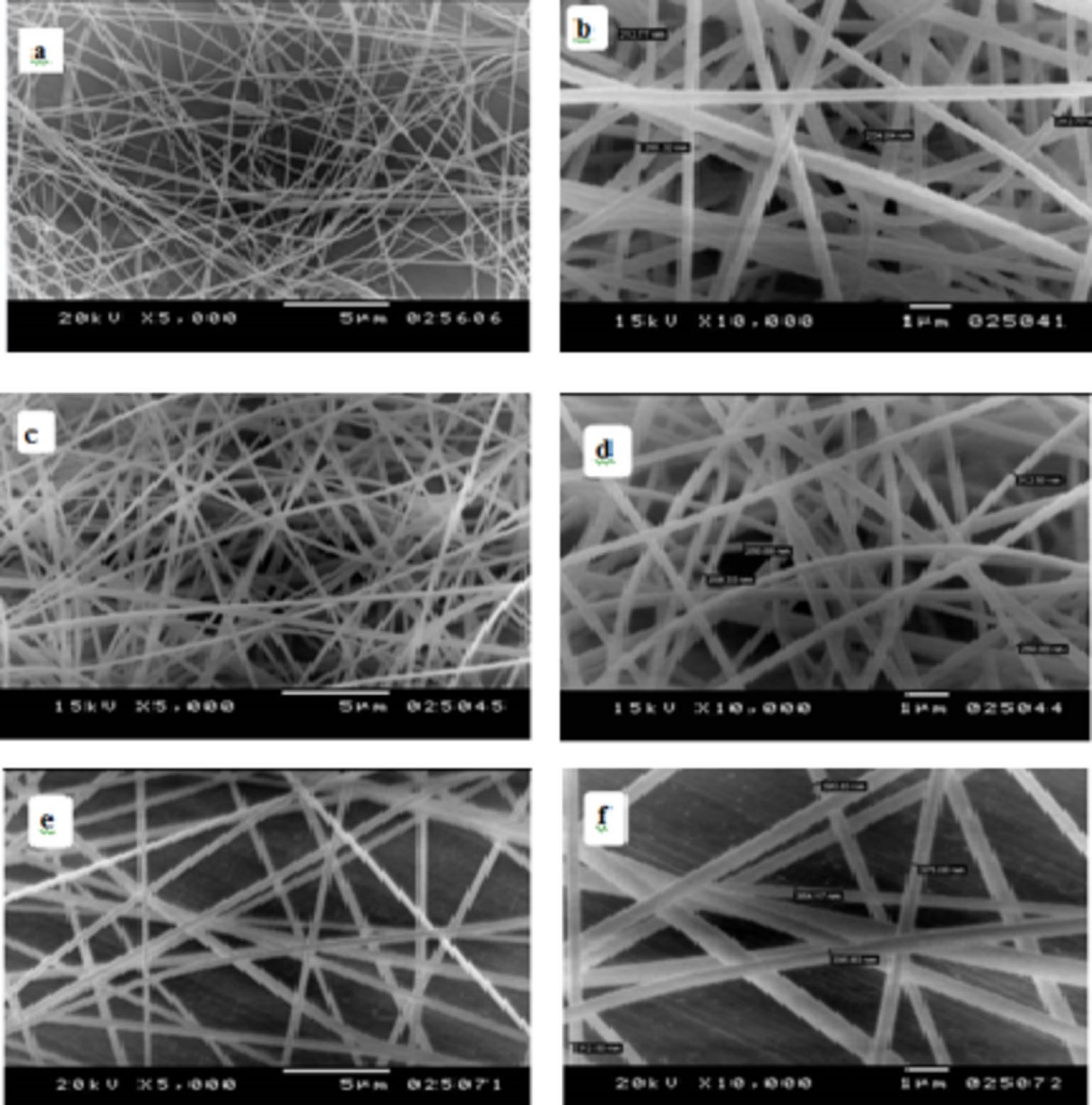
Show the SEM micrograph of PAN nanofibers (a, b) PAN 7.0 wt%, (c, d) PAN 10.0% and (e, f) 13.0wt%.

Figure 5 illustrates the SEM micrograph of electrospun pure PAN NFs with different polymer concentrations (7.0, 10.0, and 13.0 wt%) dissolved in DMF. As PAN concentration increased, the morphology altered from beaded fiber to homogeneous bead-free fiber. PAN concentration solution of 10 wt% was selected to prepare a mixed solution of PAN/AgNO3. PAN/ZnO and PAN/CuO, with concentration of metal NPs (1, 2, 3 wt%). This selection based on the most homogenous solution concentration that gave us a uniformly homogenous fiber mat when incorporated with metal nano biocides that appeared like beads connected to the nanofiber membrane make the surface of fibrous appeared rougher as result of combination between PAN and metal NPs. Those results are matched with that obtained by Shahabad et al. 2015, Huang et al., 2003^38,39^.

### SEM and EDX of Nanocomposites Fibers

PAN nanofibers containing AgNO3 were exposed to UV lamp with wavelength 254 nm and power 20 W (Cnlight China). (for 3 h, distance 20 cm) in order to induce the synthesis of silver clusters in areas adjacent to and at the surface of the fibers. Ag NPs were formed in PAN nananofibers through two steps; The initial reduction of Ag^+^ ions could occur via the oxidation reaction with DMF at room temperature, according to the following mechanism^40^:

2 $$HCONMe2\,+\,2A{g^+}+{\text{ }}H2O{\text{ }} - - - - - - - - - - - - - 2A{g^0}+Me2NCOOH\,+\,2{H^+}$$

**Fig. 6:**
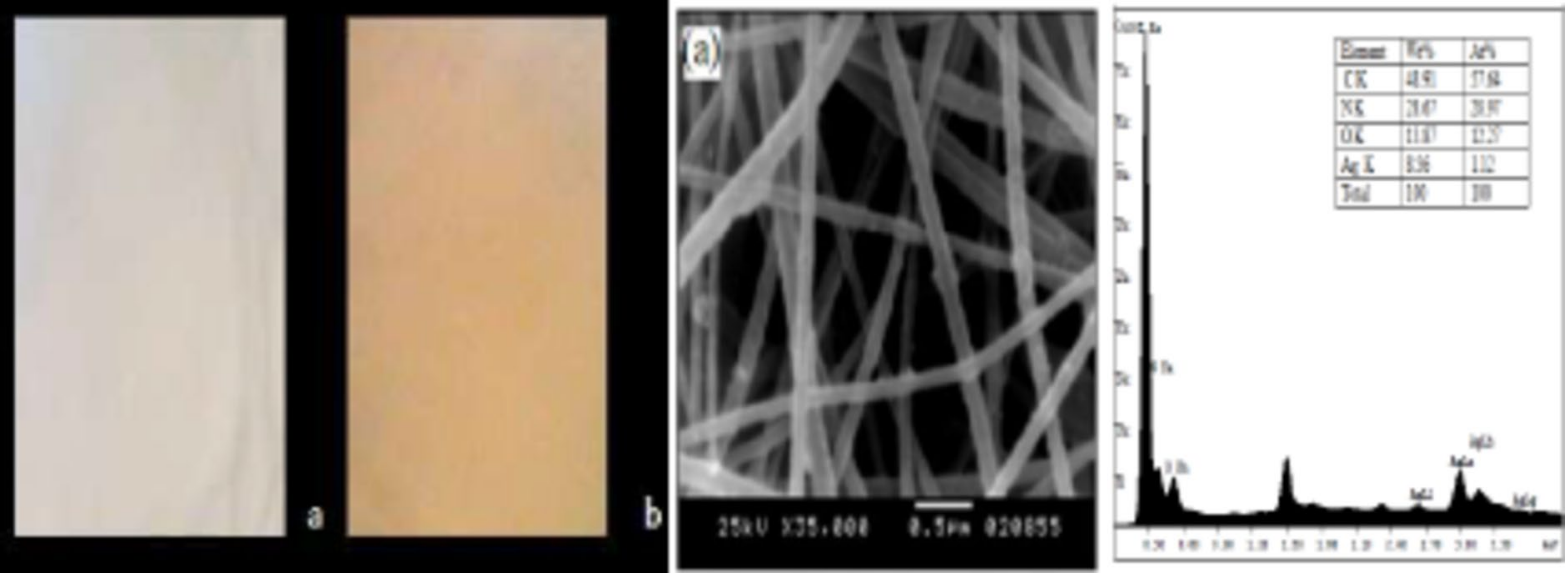
Photographs of PAN nanofibrous web (**a**) without Ag NPs and (**b**) PAN NFs loaded with Ag NPs after exposure to UV irradiation. SEM microstructures of PAN (10 wt%) loaded Ag NPs, and EDX of PAN NFs loaded with Ag NPs.

After exposure of PAN nanofibers to UV irradiation (254 nm for 3 h), the fibrous mat formed was initially pale yellow, after UV exposure, however the fiber mat had a yellow brown coloration. The change in color is due to the so called surface Plasmon resonance (SPR) phenomenon which occurs when light is reflected off thin metal film or NPs^41^. Silver nanocrystals are formed on and throughout the fiber; the silver nanoparticles are individual entities in the PAN nanofiber as shown in Fig. 6. Possible interactions between the Ag NPs and the cyano nitrogen of PAN molecules and/or the amino-nitrogen of DMF are, to some extent, effective enough to prevent the agglomeration of the as-formed particle and, at the same time, are responsible for the rather well distribution of the as-formed particles within the solutions. This method inhibits the agglomeration of the Ag NPs. The formation of AgNPs within the nanofibers was confirmed by EDX; a distinctive energy peak at around 3 KeV which is characteristic to silver was obtained indicating the presence of Ag NPs.

PAN–ZnO and PAN-CuO nanocomposites fibers with different percentage of green synthesized ZnO and CuO nanoparticles loadings (1.0, 2.0 and 3.0 wt%) were fabricated. Uniform nanocomposite fibers were fabricated from ZnO and CuO nanoparticles (1.0, 2.0 and 3.0 wt%) suspended in a PAN (10.0 wt%) DMF solution (Supplementary Figures S1-S4). However, the challenges of particle dispersion and agglomeration were encountered during electrospinning, when the ZnO and CuO nanoparticle loading increased more than 3%. The net result is a high viscosity, which prevents a continuous polymer solution jet and subsequent fiber formation. Furthermore, ZnO and CuO nanoparticles with polar surfactant on the surface carry more charges, which result in an increased electrostatic repulsion, which favors the formation of fibers with smooth surfaces. The existence and the amount of the NPs within the fiber mats were further evaluated by EDX.

**Fig. 7:**
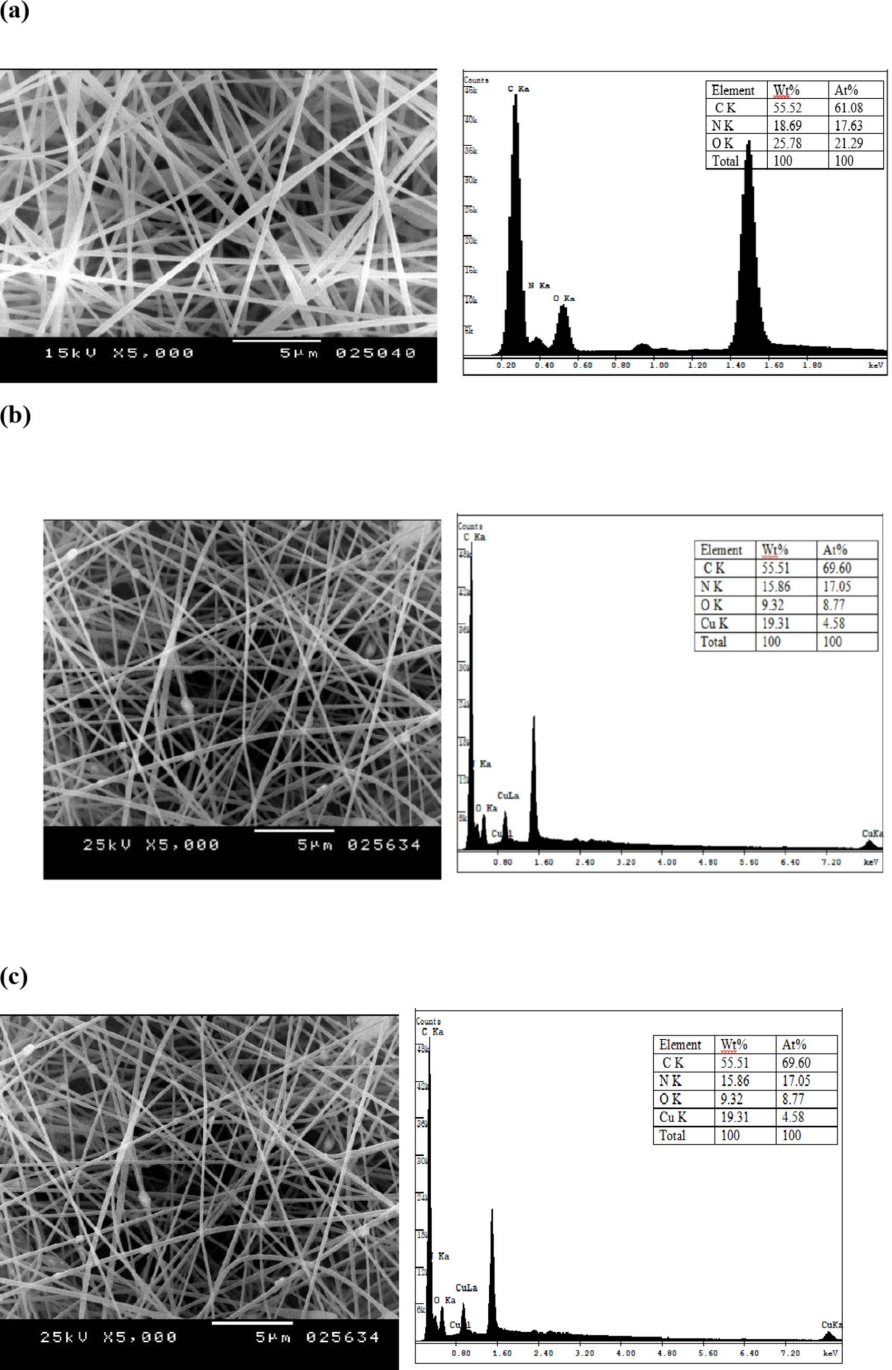
**(a)** SEM images of PAN NFs with 10 wt% (left) and its EDX (right). **(b)**: SEM images of PAN/ZnO NFs with 3% ZnO NPs (left) and its EDX (right). **(c)**: SEM images of PAN/CuO NFs with 3% CuO NPs (left) and its EDX (right).

EDX analysis of all samples revealed that the presence of considerable amounts of C, N, and O which confirmed that the obtained nanofiber mats is composed of PAN (Fig. 7a). Also, it demonstrates the successful formation of ZnO/PAN and CuO/PAN hybrid nanocomposites fibers by the presence of distinctive energy peaks for Zinc and Cupper (Fig. 7b and c).

### FT-IR spectroscopy analysis

**Fig. 8:**
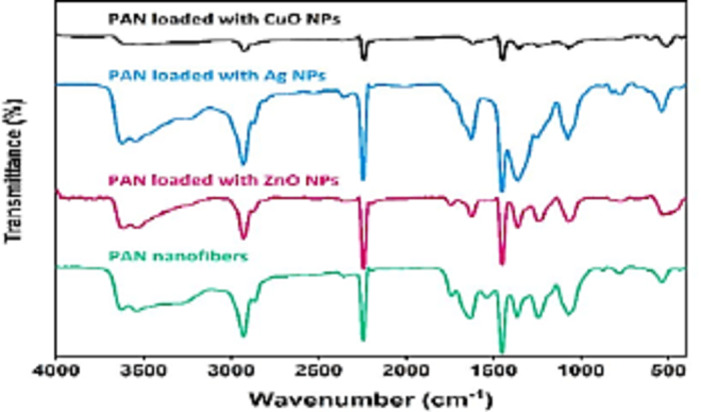
FT-IR spectra of pure PAN fibers, PAN fibers loaded with Ag NPs, PAN fibers loaded with ZnO NPs and PAN fibers loaded with CuO NPs.

The FTIR spectrum of pure PAN and its hybrid composites are shown in Fig. 8. The absorption main bands at around 2924, 1451, 1072, and 2242 and cm-1 are observed in all spectra and can be ascribed at the stretching vibration and bending vibration of the methylene (CH2–) group, C-H wagging, and stretching vibration of nitrile groups (–CN–), respectively, which are characteristics of the structure of PAN^42^. Furthermore, the weak and broad at 1660 and 3414 cm-1 assigned to C = O and stretching –OH from water adsorption on the surface of nanofiber, respectively.

The FTIR spectrum of the PAN/Ag nanocomposites fiber after UV exposure for 3 h. The peak shifting at 2265 cm-1corresponding to C-N bond towards higher wave number may be due to chemical coordination of Ag NPs with C-N bond. After the formation of Ag NPs within PAN matrix, resulting in shift to 1648 cm-1, such decrease in wave number of C = O bond may occur due to the bond weakening as a result of back bonding via partial donation of lone pair electrons from oxygen in PAN to the vacant orbital of Ag. This confirms the coordination and conjugation of embedded Ag NPs with N and O atoms of C-N and C = O bonds, respectively on the surface of PAN nanofiber^20^.

The FTIR spectrum of the PAN/ZnO nanocomposites fibers, due to the immobilization of Zn+ onto N-H and O-H functional groups, resulting in shifting in the broad band at 3000–3854 cm^− 1^ which is due to the stretching vibration of N-H and O-H primary amines (asymmetric stretch) towards lower wave number^43,44^. The sharper band at the peak at 2361 cm^− 1^ than that in pure PAN is attributed to CO_2_ absorption from air. Also, the peaks at 471 and 1654 cm^− 1^ are corresponding to Zn-O deformation and stretching vibration, respectively.

The FTIR spectrum of the PAN/CuO nanocomposites fibers, the peak at 1663 cm^− 1^ corresponds to C = O bending vibrations which indicate that the existence of the chemical bond of the polymer with the nanoparticles surface Also, the peak observed at 3335 cm^− 1^ and 1055 cm^− 1^ may be due to O-H stretching and deformation, respectively assigned to the water adsorption on the metal surface. The absorption at ~ 520 cm^− 1^ in Fig. 3-c can be the characteristic stretching vibrations of the Cu–O bond in the monoclinic CuO^45^. Therefore, the FTIR results show that the Ag/PAN, ZnO/PAN and CuO/PAN preserve the structure of PAN and the Ag, ZnO and CuO NPs are successfully composited with PAN.

### Release profile

The incorporation of nanoparticles with nanofibers enhanced the release behavior, which can inhibit the growth of bacteria. The nanofibers mats may release NPs, which accumulate inside nanofiber pores. This release progressed from the pores into the surrounding environment (water), resulting in an enhancement in the release profile. It has been reported that a steady and prolonged release of silver at a concentration level as low as 0.1 part per billion (ppb) can render effective antimicrobial activity^46^.

**Fig. 9:**
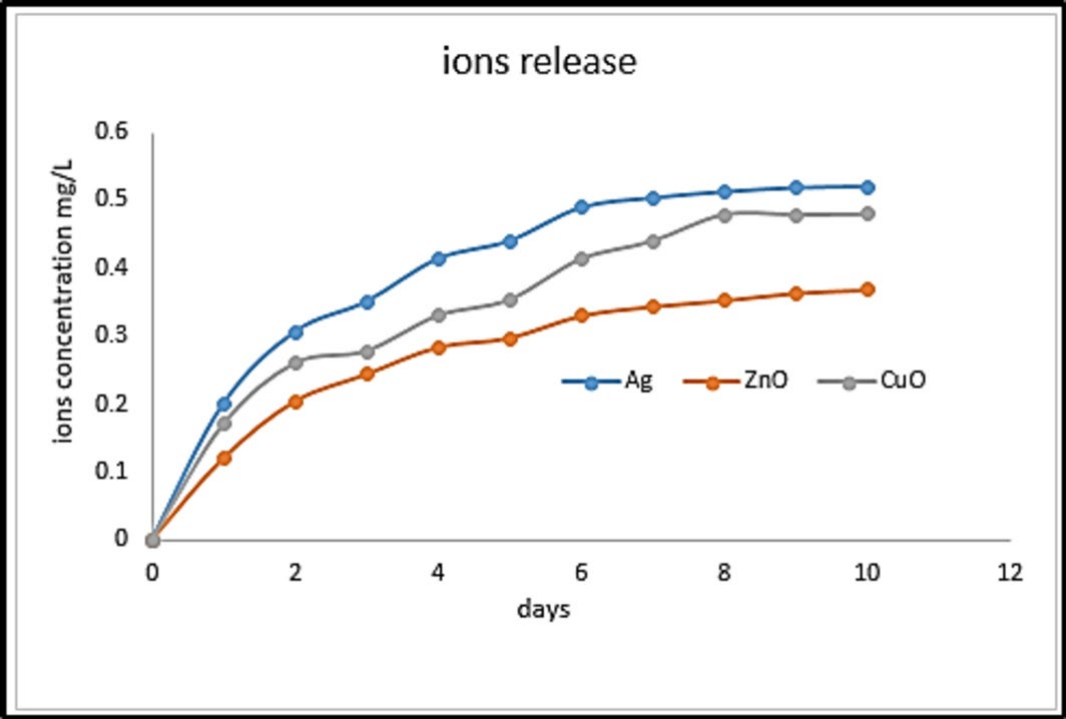
Cumulative release profiles of Ag, ZnO or CuO NPs from electrospun PAN fiber mats loaded with Ag NPs, or 3% by weight (ZnO or CuO NPs) upon total submersion in distilled water at room temperature (i.e., 25 °C ± 1 °C) (*n* = 3).

In this work, copper, zinc and silver release rates of PAN nanofibers composites were investigated in deionized water. In an aqueous environment, the nanoparticles embedded in the nanofibers are released into the solution in form of ions. The released ions were detected by AAS. Figure 9 shows the copper, zinc and silver release profiles of PAN nanofibers composites over 10 days. The release rate (i.e., the slope of the curve) is relatively high in the first few days and then decreases. The nanoparticles on the surface of nanofibers are readily available to react with water. After the initial release of surface NPs, the release process transitions to a diffusion-based release from nanoparticles embedded inside the nanofiber matrix. The release percentage over 6 days for PAN/Ag nanocomposites was 75%, and that for PAN/ZnO nanocomposites was 41%, while that for PAN/CuO nanocomposites was 68%, respectively. The ions release rate and cumulative release amount indicate that PAN nanofibers nanocomposites prepared by electrospining can release sufficient ions to exhibit sustained antibacterial activity.

The ions release and accumulated release quantity demonstrate that hybrid nanofiber produced by electrospinning may release enough ions to display prolonged antibacterial activity. As a result, these nanofibers can be recommended for extended usage in water processes, such as antimicrobial water filtrating systems. Moreover, the results demonstrated that NFs loaded with NPs could regulate the quantity of leaked NPs in deionized water over a 10-day period due to their capability to control release of metal ions.

### Mechanical properties

**Fig. 10:**
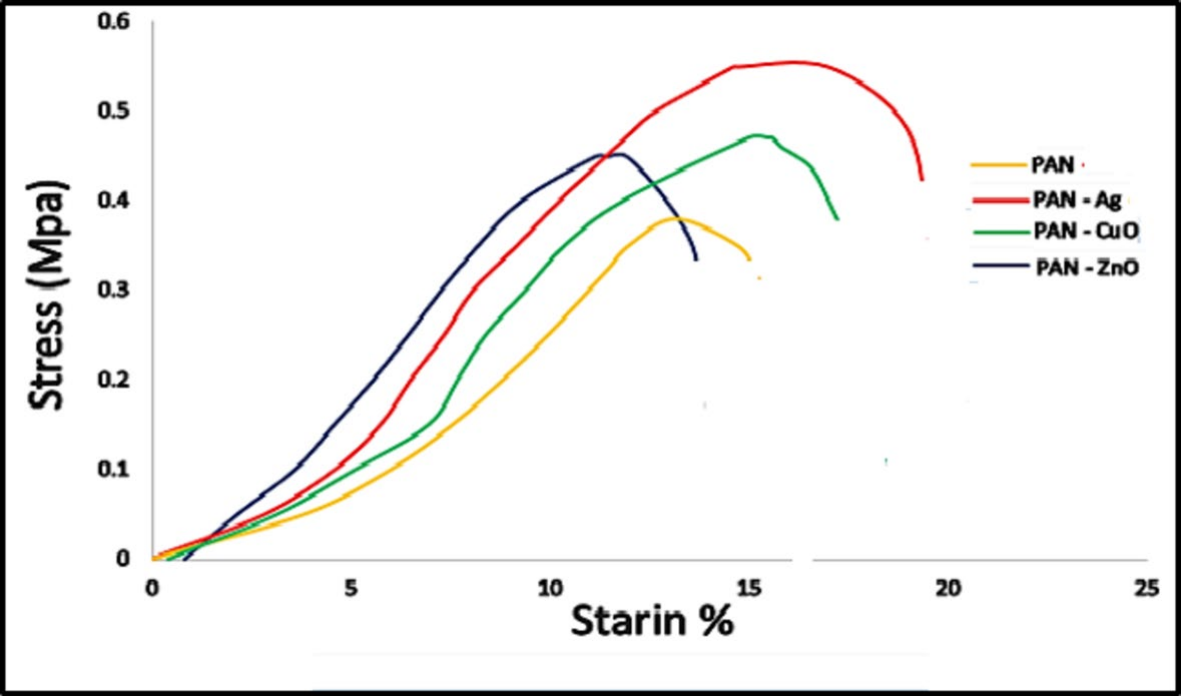
Stress – Strain curve of PAN and PAN loaded metal nanoparticles.

The mechanical properties, such as tensile strength and elongation to break, of the PAN and PAN/Ag, PAN/ZnO, and PAN/CuO nanofibers composites are shown in the stress–strain curves in Fig. 10. It is so obvious from Fig. 10 that the addition of NPs to the PAN matrix has a great influence on the mechanical properties of the composite nanofibers by increasing the tensile strength of the PAN NFs. The result showed that the plain PAN nanofibers had the ultimate tensile strength 0.34 MPs with 14% whereas PAN/Ag NPs nanofibers had 0.53 MPs and 30% elongation, and PAN/CuO NPs nanofibers had 0.45 MPa tensile strength and 28% elongation, on the other hand, PAN/ZnO NPs nanofibers had 0.42 MPa tensile strength and 20% elongation. All nanofibers loaded with NPs were larger than plain PAN nanofibers. Therefore, results demonstrated that the nanoparticles incorporation had a significantly impact on the PAN nanofibers’ tensile strength. It is desirable to generate composite nanofibers with high mechanical properties to expand the application area, such as air and water filtration^47^.

The excellent spinnability of PAN was widely recognized. However, PAN-based electrospun nanofibers continued to exhibit low mechanical characteristics. The mechanical characteristics can be improved by addition of inorganic NPs to the electrospun nanofibers^48,49^ or metal oxide nanoparticles^50,51^. The maximum value was reached when silver nanoparticles were introduced to nanofiber mats, as demonstrated in Figure (10) by the observation that all of the chosen nanoparticles might enhance the elongation at break of the nanofiber sheets. Our results were in accordance with that obtained by^52,53^, they found an obvious improvement in PVA-CuO nanofibers’ tensile strength when in comparison with PAN nanofibers. The tensile strength of PAN nanofibers was considerably enhanced by the addition of CuO nanoparticles, indicating a rise in the breaking strength of nanofibers in the existence of metal nanoparticles. The PAN/CuO nanofibers exhibited a much higher elongation at breakage in comparison to the plain PAN nanofibers. Consequently, under high tensile stresses while electrospinning processed, a greater degree of molecules orienting can be seen throughout the axis of thinner fibers, resulting in a significant shift in elastic modulus and enhanced mechanical characteristics. From other point of view, because ZnO NPs are embedded within PAN NFs, their inclusion improves the mechanical characteristics. This result was in accordance with that obtained by Zhan, et al. (2022)^54^.

### The antibacterial effect of hybrid PAN nanofibers with nanoparticles

**Fig. 11:**
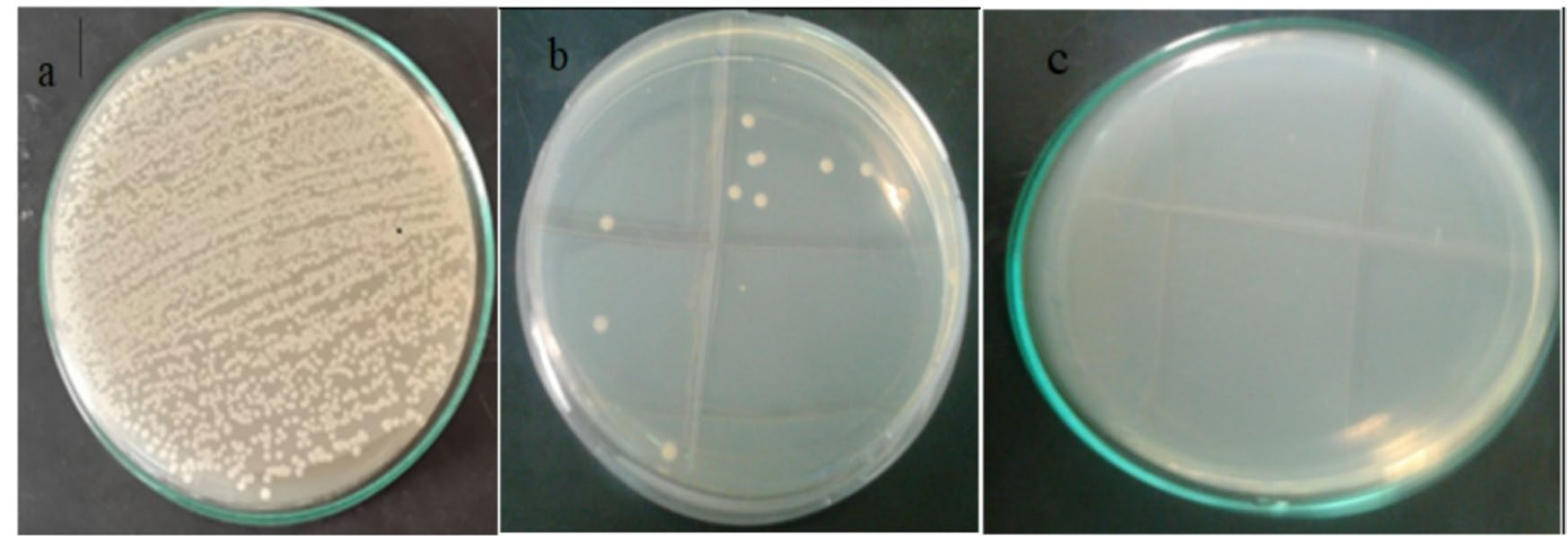
Growth control of *E. coli* grown on Muller-Hinton Agar plate. The initial CFU count with plain membrane (**a**), CFU count of bacteria separated using PAN/Ag hybrid composites after 24 h (**b**), no bacteria were found in the filtrate (**c**).

Fig. 11 Shows the effect of nanofibers impregnated with Ag NPs towards E. coli after incubation time of 24 h. Bacteria separated with plain membrane (a), A marked decrease in CFU of bacteria separated on the membrane after 24 h (Fig. 11b). Almost no bacteria were found in the filtrate (Fig. 11c). Indicating the ability of nanofiber nanofibers impregnated with Ag NPs to separate and kill bacteria found in contaminated water.

**Fig. 12:**
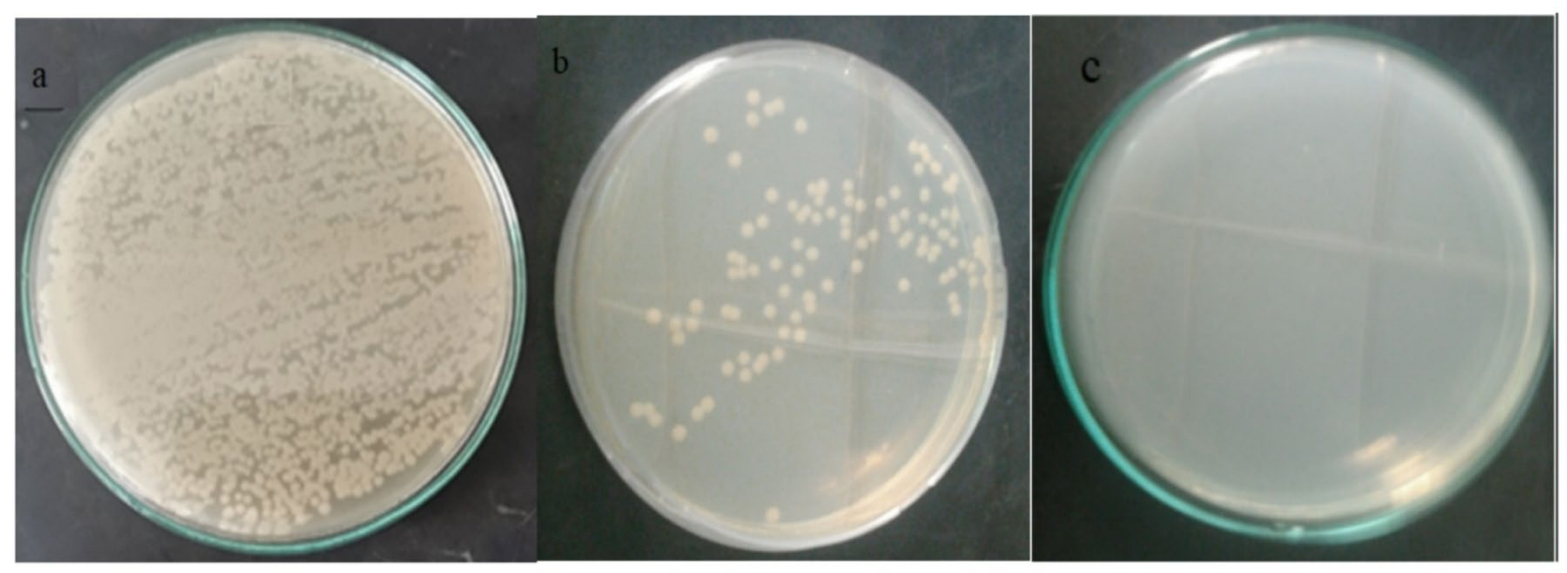
Growth control of *E. coli* grown on Muller-Hinton Agar plate with hybrid PAN nanofibers incorporated with ZnO NPs (3%wt). The initial CFU count of bacteria with plain membrane (**a**), CFU count for bacteria separated on the membrane after 24 h (**b**), no bacteria were found in the filtrate (**c**).

Figure (12) shows the effect of nanofibers impregnated with ZnO NPs against *E. coli* after incubation time 24 h. Bacteria separated with plain membrane (a), A marked decrease in CFU count of bacteria separated on the membrane after 24 h (b). Almost no growth in the filtrate (c). Indicating the ability of nanofiber nanofibers impregnated with ZnO NPs to separate and kill bacteria found in contaminated water.

**Fig. 13:**
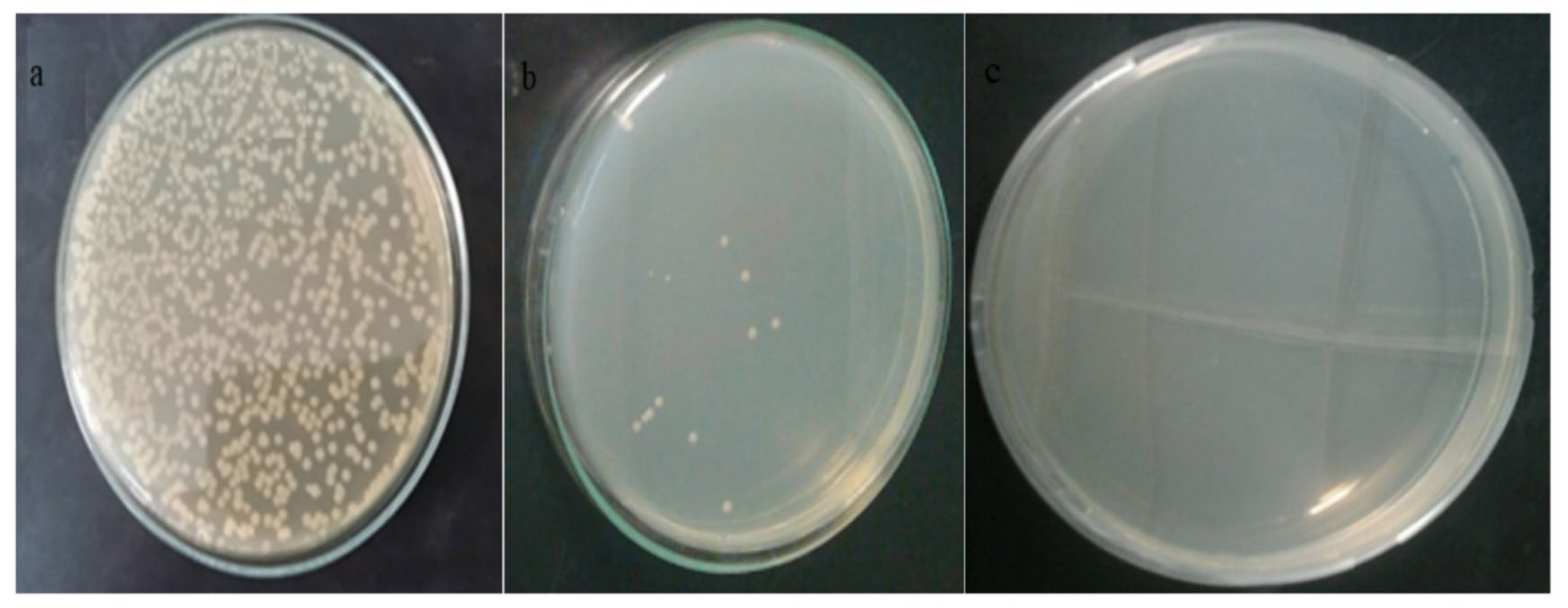
Growth control of *E. coli* grown on Muller-Hinton Agar plate with hybrid PAN nanofibers incorporated with CuO NPs (3%wt). The initial CFU count of bacteria with plain membrane (**a**), CFU count for bacteria separated on the membrane after 24 h (**b**), no bacteria were found in the filtrate (**c**).

Figure (13) shows the effect of nanofibers impregnated with CuO NPs against *E.coli* after incubation time of 24 h. A marked decrease in CFU count of bacteria separated on the membrane after 24 h. Almost no growth after 24 h. Indicating the ability of nanofiber nanofibers impregnated with CuO NPs to separate and kill bacteria found in contaminated water.

**Fig. 14:**
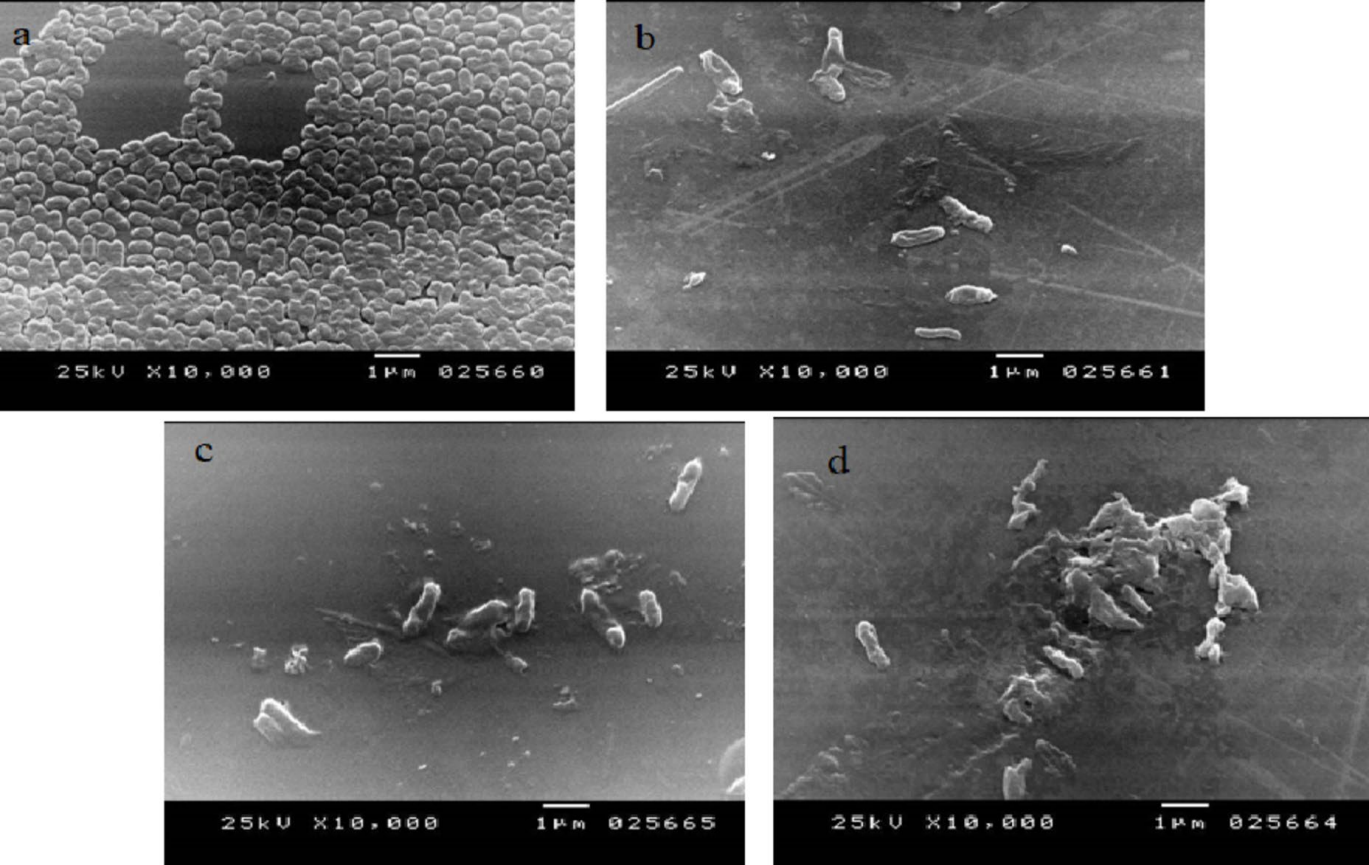
SEM image of *E. coli* bacteria cells separated by nanofiber membrane without nanoparticles (a), *E. coli* bacteria cells separated by PAN nanofibers loaded with Ag NPs (b), *E. coli* bacteria cells separated by PAN nanofibers loaded with CuO NPs, *E. coli* bacteria cells separated by PAN nanofibers loaded with ZnO NPs. Bacteria were left contact with membrane for 24 h.

To confirm the role of nanofiber membrane hybrid as antibacterial membrane, interaction of nanoparticles loaded on the nanofibers with bacterial cells was studied by SEM. To examine the interaction of nanoparticles with bacterial strains, cells separated on the nanofibers after filtration were collected to be examined by SEM. Control experiment was conducted in absence of nanoparticles. The separated cells after 24 h were prepared for observation by scanning electron microscopy (SEM, Joel, JSM-6360 LA-Japan), Figure (14).

Ag, CuO and ZnO NPs incorporated in PAN nanofibers affect bacteria along two major lethal pathways, which are related to each other and in many cases occur simultaneously: (1) the nanoparticles can release metal ions such as silver ions, zinc ions or copper ions, which are toxic to microorganisms. These ions can disrupt the cellular structures, interfere with metabolic processes, and cause oxidative stress, leading to the inactivation or death of the microorganisms. (2) production of reactive oxygen species (ROS)^55^. When NPs bind electrostatically to the bacterial cell wall and bacterial membranes, alteration of membrane potential, membrane depolarization, and loss of integrity occur which, in turn, result in an imbalance of transport, impaired respiration, interruption of energy transduction and/or cell lysis, and eventually cell death^55,56^. The high concentrations the ROS lead to cell death and at low doses cause severe DNA damage and mutations^57^. Several other effects of NPs include direct inhibition of specific essential enzymes, induction of nitrogen reactive species (NRS)^57,58^, and induction of programmed cell death^59^. This may explain our results that PAN nanofibers can separate bacteria from contaminated water, this is because the small pore size in the nanofibers. The presence of Ag, ZnO or CuO NPs impregnated in the membrane render them to kill and degrade the bacteria prevent their accumulation which may block the pores of the filter.

The mechanism by which silver nanoparticles (Ag NPs) exert their antimicrobial effects on bacteria is crucial for their effective application in water disinfection^60^. Ag NPs have the ability to connect directly with the membrane of bacterial cells, rupturing it and allowing internal components to escape, which eventually results in cell death. also has the potential to cause bacterial cells to produce ROS. This ROS have the ability to harm biological components, induce oxidative stress, and trigger cell death^61^. Ag NPs may penetrate bacterial cells and interact with DNA, leading to DNA damage. Ag NPs can bind to proteins within bacterial cells, causing denaturation and dysfunction. This disrupts cellular processes and leads to cell death^62^. Ag NPs may interfere with the electron transport chain in bacterial cells, disrupting energy production by mitochondria that is the power houses of the cells, this disruption causes cellular dysfunction and changes membrane permeability ultimately leads to cell death. These mechanisms highlight the multifaceted ways in which Ag NPs can target and eliminate bacteria, making them potent agents for water disinfection and antimicrobial applications^63^.

Zinc Oxide Nanoparticles (ZnO NPs) and their antibacterial effects on microorganisms involves several mechanisms making them promising participants for various applications, including water disinfection^64^. Within bacterial cells, ZnO NPs generate reactive oxygen species (ROS) include hydrogen peroxide and superoxide radicals. These ROS make an oxidative stress, which destroy lipids, proteins, and DNA within the cell and finally kills the bacteria. ZnO NPs interact with bacterial membrane proteins, disrupting membrane integrity. This interaction may lead to the formation of nanoparticle-protein complexes on the bacterial membrane, causing structural damage and permeabilization of the membrane. Thus, this results in leakage of cellular contents and cell death^65^. ZnO NPs have the power to directly damage bacterial cell membrane integrity. This results in cell lysis and death^56^. ZnO NPs release zinc ions (Zn²⁺) into the surrounding environment. These zinc ions can penetrate bacterial cells and interfere with various cellular processes, including enzyme activity, metabolic pathways, and DNA replication. Ultimately, this interference leads to bacterial cell death^66^.

Khatoon et al.,. 2023^67^ demonstrated three main approaches have been used to study the precise mechanism correlated to CuO NPs’ antibacterial activity toward bacteria (both gram positive and negative). These approaches include the production of damaging free radicals and reactive oxygen species (ROS) when CuO NPs dissociate, which results in the production of Cu2 + ions, and direct CuO NPs-bacterial association. Bacterial mortality results from irreversible damage caused by direct interaction of CuO NPs with the bacterium. This pathway allows the migration of nanosized CuO particles across the bacterial cell membrane (by direct diffusion or by endocytosis) because the functioning CuO NPs attached to the bacterial membrane either by molecular interactions or electrostatic connections. CuO NPs interact and attach permanently with nucleic acid molecules when entering the cytoplasm, causing the native helix structure to disorient and prevent DNA replication and synthesis. Additionally, contacts and binds with proteins and enzymes linked to essential metabolic processes as well, disturbing the bacteria’s metabolic equilibrium and eventually leading to bacterial cell death.

## Materials and methods

### Collection of water samples

One hundred different house-hold drinking tap water samples were collected from ten districts of Alexandria (Berg El Arab, El Dekhela, Abdotalat, Abo Youssef, El-Hanofel, El Betach, El Max, El Kabary, El Maferaza, Kelo 21) during summer of 2022, 10 water samples from each. We take permission to collect water samples from different household. Water samples were tested by Membrane filtration method, using 0.45 μm pore-size cellulose nitrate filter (Sartorius). The filters were cultured on: M-endo agar, M-enterococcus agar, bile esculin agar, blood agar, and MacConkey agar. All plates were incubated aerobically at 37 °C for 48 h. Colonies were provisionally identified by Gram stain and conventional biochemical reactions. Bacteria of clinical significance was stored at -20 °C in Luria Bertani broth supplemented with glycerol until needed for subsequent work.

### Preparation of Ag NPs

#### Materials

Silver nitrate powder (AgNO3, 99.99%), Molecular weight: 169,87 g/mol (Sigma Aldrich), UV lamp with wavelength 254 nm and power 20 W (Cnlight China), Glass wares (Conical flask 25 ml, Beaker 25 ml, Glass rods). All glass wears were cleaned in aqua regia (3 parts HCl, 1part HNO_3_), rinsed with deionized water, and then oven dried, Sterile forceps, Hotplate with magnetic steering and Magnet, Deionized water.

### Method

Ag NPs were prepared through photoreduction of AgNO_3_ solution using UV irradiation^68^; silver solution (3 mM) contained in sterile glass Petri dishes was exposed to UV lamp with wavelength 254 nm and power 20 W (Cnlight China), for 3 h at distance 20 cm in order to induce the synthesis of silver clusters. The prepared Ag NPs was lyophilized using lyophilizer **(**ALPHA 1–2/ LD PLUS**)** and kept in a dehydrated form (powder form) and stored at 4 ͦ C for further use. The growth of nanoparticles was monitored by UV-vis spectrophotometer and complemented with characterization using Transmission Electron Microscopy, X-ray Diffraction analysis.

### Green synthesis of ZnO NPs and Cu ONPs

#### Materials

Fresh *Rhus coriaria* fruits (Sumac) were obtained from local market in Alexandria, Egypt. Zinc acetate dihydrate (Zn (C_2_H_3_O_2_)_2_.2H_2_O, 99.0%), copper sulfate 5-hydrate (CuSO_4_·5H_2_O) and sodium Hydroxide (NaOH, pellets) was purchased from (Sigma-Aldrich, Germany).

### Method

#### Preparation of *R. coriaria* fruit aqueous extract

Fresh *R. coriaria* fruits were thoroughly washed with running tap water, followed by double-distilled water to remove any impurities. Then dried overnight in the dark at room temperature, the fruits were finely ground using a domestic blender. Then, 7.5 g of fruit powder was transferred into a flask containing 50 mL of double-distilled water, sealed, and stirred on a magnetic stirrer at 60 °C for 2 h. The solution was left at room temperature for another 24 h to ensure effective extraction of phytocompounds. Subsequently, it was subjected to centrifugation at 5000 rpm for 10 min, the supernatant was filtered using Whatman filter paper grade 1, and the resultant pale red filtrate was used for green synthesis of ZnO NPs and CuO NPs.

#### Green synthesis of ZnO NPs

Green synthesis of ZnO NPs was performed using Sumac extract by us as mentioned previously^69^. In this procedure, 50 mL of an aqueous solution of 0.1 M zinc acetate dihydrate (Zn (C_2_H_3_O_2_)_2_.2H_2_O) was prepared under moderate-speed stirring at room temperature. Then, 25 mL of sumac extract was added in a dropwise manner into the resultant aqueous solution, and the mixture was then heated at 70 °C for 2 h. Dispersed particles was observed with a notable gradual change in color to yellowish gray, indicating the formation of ZnO NPs. The pH of the reaction was adjusted to 9 using Na OH solution (1 M). Subsequently, the beaker was sealed and placed on a magnetic stirrer with a speed of 400 rpm at 70 °C for 2 h. Afterward, the solution was left to settle and placed into centrifugation at 10,000 × g for 10 min. The supernatant was then removed, and the precipitate was washed multiple times with double-distilled water, followed by ethanol, and left to dry in an oven at 80 °C overnight. Eventually, the dried powder was ground using an agate mortar and pestle and transferred into a glass vial.

#### Green synthesis of CuO NPs

To the best of knowledge, this is the first time to use of sumac fruits extracts as a bio-reducing agent for synthesis of CuO NPs. CuSO_4_.5H_2_O was used as the precursor, 50 mL of an aqueous solution of 0.4 M copper sulfate 5-hydrate (CuSO_4_.5H_2_O) was prepared under moderate-speed stirring at room temperature. An equal volume of fresh fruit extract of sumac was added into the prepared copper sulfate solution; the mixture was then heated at 80 °C for 2 h. The pH of the reaction was adjusted to 9. The solution color changed from blue to pale yellowish-green, and on vigorous stirring, the solution changed its color to dark brownish-green indicating the formation of CuO NPs. The solution was centrifuged for 20 min at 10,000 rpm, the supernatant was then removed, and the precipitate was washed multiple times with double-distilled water, followed by ethanol, and left to dry in an oven at 80 °C overnight. Eventually, the dried powder was ground using an agate mortar and pestle and transferred into a glass vial. The growth of nanoparticles (ZnO NPs and CuONPs) was monitored by UV-vis spectrophotometer and complemented with characterization using Transmission Electron Microscopy, X-ray Diffraction analysis.

### Preparation of PAN nanofiber membrane and PAN hybrid composite membranes

#### Reagent

Polyacrylonitrile (PAN, Mw 150,000 g/mol, Sigma-Aldrich) and N, N’- dimethylformamide (DMF, Sigma-Aldrich), Ag NO3, ZnO and CuO NPs.

### Preparation of polymer solutions

Different concentrations of PAN solutions (7wt%, 10 wt% and 13 wt%) were prepared for the electrospinning experiments. The PAN solution was prepared by dissolving PAN in DMF at 80 °C with continuous stirring (very low stirring) for 6 h. The obtained transparent homogenous solution was then cooled at room temperature.

### Preparation of nanocomposite solutions

Fibrous membranes loaded with ZnO or CuO NPs, were prepared from 10% w/v polyacrylonitrile (PAN) solutions; as it is the best characterized one. Firstly, green synthesized ZnO or CuO NPs powder was firstly dispersed in DMF with percentage (1, 2 and 3% by weight of PAN), according to our previous study^20^, under continuous magnetic stirring. After complete dispersion of NPs, PAN was then added gradually, the solution was stirred at 80 °C for 6 h. The obtained homogenous solution was then cooled at room temperature.

Fibrous membranes loaded with Ag NO3 were prepared from 10% w/v polyacrylonitrile. AgNO3 was dispersed in DMF in the amounts of 1, 2 and 3% by weight of PAN. DMF was used as both the solvent for PAN and reducing agent for Ag ions. After complete dispersion of AgNO3, PAN was then added gradually, the solution was stirred at 80 °C for 6 h. The obtained homogenous solution was then cooled at room temperature.

## Fabrication of nanofibers composites membranes

All mixtures were homogeneous, transparent, and free from all air bubbles or precipitates with suitable viscosity for spinning. Both the plain polymer solution and NPs suspended polymer solution were used for nanofibers composites fabrication using electrospinning, separately. Each solution (PAN solution, PAN/ZnO, PAN/CuO or PAN/AgNO3) was collected separately into a 10 ml syringe equipped with a 24-stainless steel blunted tip needle. PAN nanofibers were fabricated using the electrospinning technique in an air-conditioned laboratory. The process conditions were kept at an ambient temperature of 22 °C and relative humidity of < 65%. The syringe was fixed on an electric syringe pump set to maintain a constant feed rate of 1.5 ml/h. A high voltage power supply (Gamma high voltage, Inc., USA) was employed to apply positive charge to the needle, and a grounded metal plate covered with aluminum foil served as the collector. Electric potential and distance to collector was fixed at 20 kV and 15 cm, respectively. PAN nanofibers containing AgNO_3_ were exposed to UV irradiation using UV lamp with wavelength 254 nm and power 20 W (Cnlight China) at a distance 20 cm for 3 h in order to complete silver ion reduction in the nanofibers to Ag NPs.

### Characterization of hybrid nanofiber membrane composites

The morphology of the electrospun PAN nanofibers and that containing Ag, ZnO and CuO NPs were observed using a scanning electron microscope (SEM, JOEL, JSM-6360 LA-Japan) after gold coating. The average diameter of PAN nanofibers was obtained by analyzing SEM images using an image analysis program. The presence of Ag, ZnO and CuO NPs in PAN nanofibers were confirmed through energy dispersive spectroscopy (EDS). The surface functional groups of the prepared NFs have been investigated by Fourier Transform Infrared Spectrophotometer (Shimazdu IR Prestige-21, Japan) in the mid-IR region (4000–400 cm − 1) and a standard technique with KBr pellets.

### Release profile

Release Profile was studies by using Atomic Absorption Spectrometry (AAS, Perkin- Elmer AA300, Thermo Scientific ICE 3500). The release profile over a period of 10 days was studied by dipping 0.1 g of nanofibers in a container containing 20 mL of deionized water by shaking at room temperature (i.e., 25 ± 1 °C). The container was sealed and agitated to insure complete immersion of the nanofibrous mat. At a specified immersion time point ranging between 0 and 10 days, the amount of the released ions in the releasing medium was quantified using AAS. The cumulative release profiles of NPs were expressed based on the unit weight of the specimens. The amount of NPs loaded into nanofibers was measured by dissolving the 0.01 g nanofibers in 5 mL concentrated nitric acid (HNO3) which was completed to 1000 ml using deionized water, the obtained clear solution was supplied to AA spectrometry. The release profile was presented as a percentage based on the below formula:

3 $$Release\: \% = \: Mi/ Mt$$

where ***Mi*** is the amount of NPs released after 10 days, ***Mt*** is the total amount of NPs loaded in PAN nanofibers.

#### Mechanical properties (tensile strength)

The mechanical properties of PAN and PAN loaded with Ag NPs, ZnO NPs and CuO NPs were assessed using Tinius Olsen Testing Machine (Co., Inc. United State of America), with force 1 N, under a cross-head speed of 5 mm/min at room temperature. In accordance with ASTM D-638, the nonwoven nanofibers were prepared by cutting the nanofiber membrane into rectangle shape has dimension of length 5 cm, width 1 cm, the thickness of the samples was measured at four random positions by a micrometer. Tensile properties of PAN loaded with NPs nanofibers were measured using at least three specimens per samples. Stress and strain were calculated by Formulas (4) and (5) as mentioned below:

4 $$\:\mathrm{S}\mathrm{t}\mathrm{r}\mathrm{e}\mathrm{s}\mathrm{s}\:\left(\mathrm{S}\mathrm{t}\mathrm{r}\mathrm{e}\mathrm{n}\mathrm{t}\mathrm{h}\right)=\frac{\mathrm{F}\mathrm{o}\mathrm{r}\mathrm{c}\mathrm{e}\:\left(\mathrm{N}\right)}{\mathrm{A}\mathrm{r}\mathrm{e}\mathrm{a}\:\left(\mathrm{w}\mathrm{i}\mathrm{d}\mathrm{t}\mathrm{h}\:\mathrm{x}\:\mathrm{t}\mathrm{h}\mathrm{i}\mathrm{c}\mathrm{k}\mathrm{n}\mathrm{e}\mathrm{s}\mathrm{s}\right)}\:\:\:$$

5 $$\:\mathrm{S}\mathrm{t}\mathrm{r}\mathrm{a}\mathrm{i}\mathrm{n}\:\left(\mathrm{E}\mathrm{l}\mathrm{o}\mathrm{n}\mathrm{g}\mathrm{a}\mathrm{t}\mathrm{i}\mathrm{o}\mathrm{n}\right)=\:\frac{\mathrm{C}\mathrm{h}\mathrm{a}\mathrm{n}\mathrm{g}\mathrm{e}\:\mathrm{i}\mathrm{n}\:\mathrm{l}\mathrm{e}\mathrm{g}\mathrm{t}\mathrm{h}\:(\varDelta\:\mathrm{l})}{\mathrm{I}\mathrm{n}\mathrm{i}\mathrm{t}\mathrm{i}\mathrm{a}\mathrm{l}\:\mathrm{l}\mathrm{e}\mathrm{n}\mathrm{g}\mathrm{t}\mathrm{h}\:\left(\mathrm{l}\right)}\:\mathrm{x}100$$

## Antibacterial efficiency of nanofibers hybrid membrane using filtration unit

**Fig. 15:**
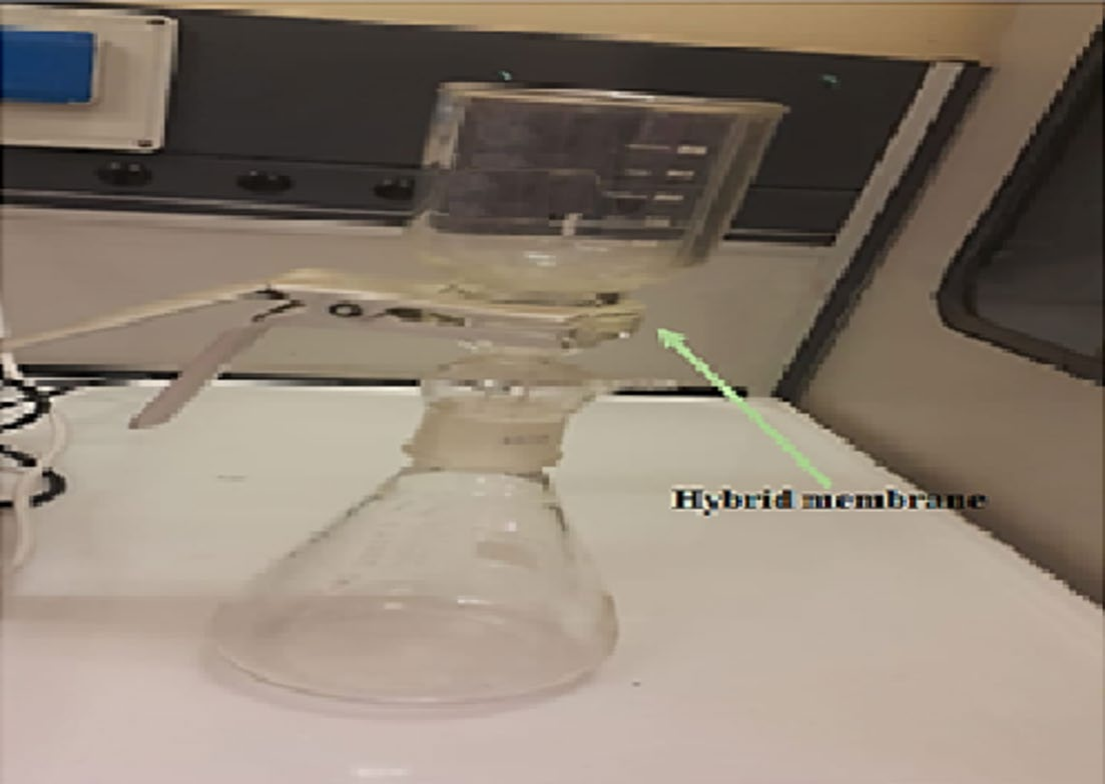
The prototype membrane filtration unit used for water disinfection. The hybrid membrane is placed in the middle of the mouth flask and the catcher where they are adjacent to each other by a filter. Contaminated water was slowly poured down into the catcher. After each filtration, the membrane is washed to remove any microbe inside the membrane to be studied.

The isolated *E. coli* was selected as indicators of fecal contamination of water. Nutrient broth was used as the growing medium. Bacteria were grown aerobically in nutrient broth at 37 °C for 24 h. The cultures were centrifuged, and the Bactria were washed and suspended in distilled water, reaching a final concentration of 10^5^ to 10^6^ CFU/mL. The antibacterial efficiencies of nanofiber hybrid membranes (Ag loaded, ZnO loaded or CuO loaded membranes) were evaluated by contact test by immobilizing the nanofibers onto ceramic water filter candle filters of the filtration unit (Fig. 15). The test water sample was prepared by inoculating 10^6^cells/ml *E. coli* into 200 ml of distilled water. Water samples were then filtered through 0.22 μm filters, with either plain PAN nanofibers, or PAN nanofibers containing the nanoparticles (Ag NPs, ZnO NPs or CuO NPs), immobilized on the filter surface of the filtration unit. The filter pore size of 0.22 μm excluded bacterial cells, causing separated bacteria to remain in contact with the nanofibers. After filtration, the viability of bacteria remaining on the nanofibers with hybrid membrane nanoparticles was investigated, by determining the viable cells recovered from the nanofibers. This was done by washing of nanofibers after contact times of 24 h. Washed bacterial samples were plated onto solid nutrient agar plates then incubated overnight at 37˚C, CFU was visualized and photographed.

## Conclusion

Clean water is essential for human health. It is a precious resource that requires effective management, conservation, and equitable distribution to ensure a healthy and sustainable future for all. Green synthesis of NPs represents a straightforward, eco-friendly, and cost-effective method. Ag NPs have much more antimicrobial properties than CuO and ZnO NPs due to their small size. Electrospinning technique is a simple technique for manufacturing of antimicrobial membranes for water treatment. Electrospun hybrid membrane of PAN nanofiber incorporated with Ag NPs, ZnO NPs or CuO NPs increases disinfection properties of the hybrid membrane. Such hybrid membrane preventing biofouling in the membrane and gave the clearest filtrate. Loading of nanoparticles increased the mechanical properties of PAN nanofibers. The inhibition of growth of *E. coli* by nanofibers incorporated with Ag, ZnO or CuO nanoparticles indicate that it could ultimately be used as efficient antibacterial membranes for water disinfection and filtering.

The key limitation in this study was its confinement of antibacterial evolution of the hybrid nanofibers membranes using contact test. Future study should measure the antibacterial activity of green synthesized nanoparticles before and after loading on the polymer nanofibers. Future investigation should prioritize to measure the filtration parameters such as flux rate, permeability, rejection efficiency. In the future study, the durability of the hybrid membranes to be used in water filtration without fouling should be studied.

## Electronic Supplementary Material

Below is the link to the electronic supplementary material.

Supplementary Material 1

Supplementary Material 2

Supplementary Material 3

Supplementary Material 4

## Data Availability

All data generated or analyzed during the current study are available from the corresponding author upon reasonable request.

## References

[1] Saravanan, A. et al. A review on biosynthesis of metal nanoparticles and its environmental applications. *Chemosphere* **264**, 128580 (2021). 33059285 10.1016/j.chemosphere.2020.128580

[2] Anatolia, L. et al. *H*ow to Cite Efforts to Improve Clean Water Quality to Support Community Health Efforts to Improve Clean Water Quality to Support Community Health. *KESANS Int. J. Health Sci.* **1**, 2808–7178 (2021).

[3] Nasir, A. M. et al. A review of the potential of conventional and advanced membrane technology in the removal of pathogens from wastewater. *Sep. Purif. Technol.* **286**, 120454 (2022). 35035270 10.1016/j.seppur.2022.120454 PMC8741333

[4] Wimalawansa, S. Purification of Contaminated Water with Reverse Osmosis: Effective Solution of Providing Clean Water for Human Needs in Developing Countries. *Int. J. Emerg. Technol. Adv. Engin*. **3**, 9001 (2013).

[5] Shoshaa, R., Ashfaq, M. Y. & Al-Ghouti, M. A. Recent developments in ultrafiltration membrane technology for the removal of potentially toxic elements, and enhanced antifouling performance: A review. *Environ. Technol. Innov.* **31**, 103162 (2023).

[6] Qiongjie, W. et al. Effects of biofilm on metal adsorption behavior and microbial community of microplastics. *J. Hazard. Mater.* **424**, 127340 (2022). 34607028 10.1016/j.jhazmat.2021.127340

[7] Joseph, T. M. et al. Nanoparticles and nanofiltration for wastewater treatment: From polluted to fresh water. *Environ. Res.* **238**, 117114 (2023). 37716387 10.1016/j.envres.2023.117114

[8] Ramezani, M. R. et al. Polyacrylonitrile/Fe(III) metal-organic framework fibrous nanocomposites designed for tissue engineering applications. *Mater. Chem. Phys.* **229**, 242–250 (2019).

[9] Xue, J. et al. Electrospinning and Electrospun Nanofibers: Methods, Materials, and Applications. *Chem. Rev.* **119** (8), 5298–5415 (2019). 30916938 10.1021/acs.chemrev.8b00593 PMC6589095

[10] Sagitha, P. et al. Recent advances in post-modification strategies of polymeric electrospun membranes. *Eur. Polymer J.* **105**, 227–249 (2018).

[11] Toriello, M. et al. *Progress on the Fabrication and Application of Electrospun Nanofiber Composites*. Membranes 10, (2020). 10.3390/membranes10090204 10.3390/membranes10090204 PMC7559347 32872232

[12] Haider, M. K. et al. Fabricating Antibacterial and Antioxidant Electrospun Hydrophilic Polyacrylonitrile Nanofibers Loaded with AgNPs by Lignin-Induced In-Situ Method. *Polymers* **13**10.3390/polym13050748 (2021). 10.3390/polym13050748 PMC7957607 33670863

[13] Liao, Y. et al. Progress in electrospun polymeric nanofibrous membranes for water treatment: Fabrication, modification and applications. *Prog. Polym. Sci.* **77**, 69–94 (2018).

[14] Khan, A. A. et al. Metal oxide and carbon nanomaterial based membranes for reverse osmosis and membrane distillation: A comparative review. *Environ. Res.* **202**, 111716 (2021). 34293311 10.1016/j.envres.2021.111716

[15] Singh, R. et al. *Nanofiltration technology for removal of pathogens present in drinking water*: Waterborne Pathogens. :463 – 89. (2020). 10.1016/B978-0-12-818783-8.00021-9. Epub 2020 Feb 14.

[16] Uddin, Z. et al. Recent trends in water purification using electrospun nanofibrous membranes. *Int. J. Environ. Sci. Technol.* **19** (9), 9149–9176 (2022).

[17] Jena, S. K. et al. Nanoremediation: sunlight mediated dye degradation using electrospun PAN/CuO–ZnO nanofibrous composites. *Environ. Pollut.* **280**, 116964 (2021). 33794417 10.1016/j.envpol.2021.116964

[18] Guan, G. et al. Antibacterial properties and mechanism of biopolymer-based films functionalized by CuO/ZnO nanoparticles against Escherichia coli and Staphylococcus aureus. *J. Hazard. Mater.* **402**, 123542 (2021). 32745874 10.1016/j.jhazmat.2020.123542

[19] Bode-Aluko, C. A. et al. Surface-modified polyacrylonitrile nanofibres as supports. *Polym. Bull.* **74** (6), 2431–2442 (2017).

[20] Shalaby, T. et al. Electrospun nanofibers hybrid composites membranes for highly efficient antibacterial activity. *Ecotoxicol. Environ. Saf.* **162**, 354–364 (2018). 30007185 10.1016/j.ecoenv.2018.07.016

[21] Ana-Maria, P. & Cadar, E. Method for Obtaining and Physico-Chemical Characterization of Collagenic Extract of Rhizostoma Pulmo from the Black Sea. *Eur. J. Med. Nat. Sci.* **5**, 48 (2022).

[22] Rao, T. L. S. K. J. & Korumilli, T. Natural Biogenic Templates for Nanomaterial Synthesis: Advances, Applications, and Environmental Perspectives. *ACS Biomaterials Sci. Eng.* **11** (3), 1291–1316 (2025). 10.1021/acsbiomaterials.4c02075 39928588

[23] Ayu, R. et al. *Microbiological Contaminants in Drinking Water: Current Status and Challenges* 233 (Water, Air, & Soil Pollution, 2022).

[24] Cui, J. et al. Electrospun nanofiber membranes for wastewater treatment applications. *Sep. Purif. Technol.* **250**, 117116 (2020).

[25] Mawaddah, M. et al. *Green synthesis of silver nanoparticles using photo-induced reduction method*. Vol. 2018. 020082. (2049).

[26] Sichani, G. N. et al. In situ preparation, electrospinning, and characterization of polyacrylonitrile nanofibers containing silver nanoparticles. *J. Appl. Polym. Sci.* **116** (2), 1021–1029 (2010).

[27] Singh, J. et al. Green’ synthesis of metals and their oxide nanoparticles: applications for environmental remediation. *J. Nanobiotechnol.* **16** (1), 018–0408 (2018). 10.1186/s12951-018-0408-4 PMC6206834 30373622

[28] Elumalai, E. et al. *Green synthesis of silver nanoparticle using Euphorbia hirta L and their antifungal activities.* (2010).

[29] Faisal, S. et al. Green Synthesis of Zinc Oxide (ZnO) Nanoparticles Using Aqueous Fruit Extracts of Myristica fragrans: Their Characterizations and Biological and Environmental Applications. *ACS Omega*. **6** (14), 9709–9722 (2021). 33869951 10.1021/acsomega.1c00310 PMC8047667

[30] Abdullah, J. A. A. et al. Eco-friendly synthesis of ZnO-nanoparticles using Phoenix dactylifera L., polyphenols: physicochemical, microstructural, and functional assessment. *New J. Chem.* **47** (9), 4409–4417 (2023).

[31] Gebremedhn, K., Kahsay, M. H. & Aklilu, M. Green synthesis of CuO nanoparticles using leaf extract of Catha edulis and its antibacterial activity. *J. Pharm. Pharmacol.* **7** (6), 327–342 (2019).

[32] Mary, A. A., Ansari, A. T. & Subramanian, R. Sugarcane juice mediated synthesis of copper oxide nanoparticles, characterization and their antibacterial activity. *J. King Saud University-Science*. **31** (4), 1103–1114 (2019).

[33] Nadaf, S. J. et al. Green synthesis of gold and silver nanoparticles: Updates on research, patents, and future prospects. *OpenNano* **8**, 100076 (2022).

[34] Narayana, A. et al. Green and low-cost synthesis of zinc oxide nanoparticles and their application in transistor-based carbon monoxide sensing. *RSC Adv.* **10** (23), 13532–13542 (2020). 35492987 10.1039/d0ra00478b PMC9051533

[35] Gnanavel, V., Palanichamy, V. & Roopan, S. M. Biosynthesis and characterization of copper oxide nanoparticles and its anticancer activity on human colon cancer cell lines (HCT-116). *J. Photochem. Photobiol., B*. **171**, 133–138 (2017). 28501691 10.1016/j.jphotobiol.2017.05.001

[36] David, A., Raj, D. & Kumar, S. Biosynthesis of copper oxide nanoparticles using Momordica charantia leaf extract and their characterization. *Int. J. Adv. Res. Sci. Eng.* **06** (03), 313–320 (2017).

[37] Sudha, V. et al. Copper oxide nanosheet modified electrodes for simultaneous determination of environmentally hazardous anions. *J. Alloys Compd.* **764**, 959–968 (2018).

[38] Shahabadi, M. et al. Effects of process and ambient parameters on diameter and morphology of electrospun polyacrylonitrile nanofibers. *Polym. Sci. Ser. A*. **57**, 155–167 (2015).

[39] Huang, Z. M. et al. A review on polymer nanofibers by electrospinning and their applications in nanocomposites. *Compos. Sci. Technol.* **63** (15), 2223–2253 (2003).

[40] Rujitanaroj, P., Pimpha, N. & Supaphol, P. Preparation, characterization, and antibacterial properties of electrospun polyacrylonitrile fibrous membranes containing silver nanoparticles. *J. Appl. Polym. Sci.* **116** (4), 1967–1976 (2010).

[41] Shalaby, T., Mahmoud, O. & Al-Oufy, A. Antibacterial silver embedded nanofibers for water disinfection. *Int. J. Mater. Sci. Appl.* **4** (5), 293–298 (2015).

[42] Chang, H. et al. Gel spinning of polyacrylonitrile/cellulose nanocrystal composite fibers. *ACS Biomaterials Sci. Eng.* **1** (7), 610–616 (2015). 10.1021/acsbiomaterials.5b00161 33434977

[43] El Essawy, N. A. et al. Green synthesis of graphene from recycled PET bottle wastes for use in the adsorption of dyes in aqueous solution. *Ecotoxicol. Environ. Saf.* **145**, 57–68 (2017). 28708982 10.1016/j.ecoenv.2017.07.014

[44] Munnawar, I. et al. Synergistic effect of Chitosan-Zinc Oxide Hybrid Nanoparticles on antibiofouling and water disinfection of mixed matrix polyethersulfone nanocomposite membranes. *Carbohydr. Polym.* **175**, 661–670 (2017). 28917915 10.1016/j.carbpol.2017.08.036

[45] Pan, Y. et al. Nitrogen-doped graphene oxide/cupric oxide as an anode material for lithium ion batteries. *RSC Adv.* **4** (110), 64756–64762 (2014).

[46] Kumar, R. & Münstedt, H. Silver ion release from antimicrobial polyamide/silver composites. *Biomaterials* **26** (14), 2081–2088 (2005). 15576182 10.1016/j.biomaterials.2004.05.030

[47] Abdel-Mottaleb, M. et al. *Preparation, characterization, and mechanical properties of polyacrylonitrile (PAN)/graphene oxide (GO) nanofibers* 27 (Mechanics of Advanced Materials and Structures, 2018).

[48] Li, X. et al. Preparation, characterization and antibacterial activity of quaternized carboxymethyl chitosan/organic rectorite nanocomposites. *Curr. Nanosci.* **9** (2), 278–282 (2013).

[49] Lv, H. et al. Electrospun PCL-based polyurethane/HA microfibers as drug carrier of dexamethasone with enhanced biodegradability and shape memory performances. *Colloid Polym. Sci.* **298**, 103–111 (2019).

[50] Sessini, V. et al. Thermally activated shape memory behavior of copolymers based on ethylene reinforced with silica nanoparticles. *Nanocomposites* **4** (2), 19–35 (2018).

[51] Zhang, Q. et al. Controllable synthesis of peapod-like TiO2@GO@C electrospun nanofiber membranes with enhanced mechanical properties and photocatalytic degradation abilities towards methylene blue. *New J. Chem.* **44** (9), 3755–3763 (2020).

[52] Fattahi, M. et al. Evaluation of the efficacy of NanoPak Mask^®^: A polyacrylonitrile/copper oxide nanofiber respiratory mask. *Mater. Today Commun.* **38**, 108129 (2024).

[53] Sarwar, M. N. et al. Electrospun PVA/CuONPs/Bitter Gourd Nanofi bers with Improved Cytocompatibility and Antibacterial Properties: Application as Antibacterial Wound Dressing. *Polymers (Basel) ***14** (7), 1361. https://dio.org/10.3390/polym14071361 (2022). 10.3390/polym14071361 PMC9002528 35406236

[54] Zhan, J. et al. Fabrication, characterization and antibacterial properties of ZnO nanoparticles decorated electrospun polyacrylonitrile nanofibers membranes. *Mater. Today Commun.* **32**, 103958 (2022).

[55] Pelgrift, R. Y. & Friedman, A. J. Nanotechnology as a therapeutic tool to combat microbial resistance. *Adv. Drug Deliv Rev.* **65** (13–14), 1803–1815 (2013). 23892192 10.1016/j.addr.2013.07.011

[56] Modi, S. K. et al. Mechanistic insights into nanoparticle surface-bacterial membrane interactions in overcoming antibiotic resistance. *Front. Microbiol.* **14**, 1135579 (2023). 37152753 10.3389/fmicb.2023.1135579 PMC10160668

[57] Pan, X. et al. Mutagenicity evaluation of metal oxide nanoparticles by the bacterial reverse mutation assay. *Chemosphere* **79** (1), 113–116 (2010). 20106502 10.1016/j.chemosphere.2009.12.056

[58] Nathan, C. & Cunningham-Bussel, A. Beyond oxidative stress: An immunologist’s guide to reactive oxygen species. *Nat. Rev. Immunol.* **13**, 349–361 (2013). 23618831 10.1038/nri3423 PMC4250048

[59] Loomba, L. & Scarabelli, T. Metallic nanoparticles and their medicinal potential. Part I: gold and silver colloids. *Therapeutic delivery*. **4** (7), 859–873 (2013). 23883128 10.4155/tde.13.55

[60] Dutta, T. et al. Antimicrobial silver nanoparticles for water disinfection: a short review on recent advances. *Nanatechnol. Environ. Eng.* **9** (1), 111–131 (2024).

[61] Kawish, M. et al. *Bactericidal potentials of silver nanoparticles: novel aspects against multidrug resistance bacteria, in Metal Nanoparticles for Drug Delivery and Diagnostic Applications* p. 175–188 (Elsevier, 2020).

[62] Li, N. et al. The uptake, elimination, and toxicity of silver nanoparticles and silver ions in single-species and natural mixed-species bacterial biofilms. *J. Water Process. Eng.* **56**, 104256 (2023).

[63] Flores-Lopez, L. Z., Espinoza-Gomez, H. & Somanathan, R. Silver nanoparticles: Electron transfer, reactive oxygen species, oxidative stress, beneficial and toxicological effects. Mini review. *J. Appl. Toxicol.* **39** (1), 16–26 (2019). 29943411 10.1002/jat.3654

[64] Raghavendra, V. B. et al. Green Synthesis of Zinc Oxide Nanoparticles (ZnO NPs) for Effective Degradation of Dye, Polyethylene and Antibacterial Performance in Waste Water Treatment. *J. Inorg. Organomet. Polym Mater.* **32** (2), 614–630 (2021).

[65] Mendes, C. R. et al. Antibacterial action and target mechanisms of zinc oxide nanoparticles against bacterial pathogens. *Sci. Rep.* **12** (1), 2658 (2022). 35173244 10.1038/s41598-022-06657-y PMC8850488

[66] Godoy-Gallardo, M. et al. Antibacterial approaches in tissue engineering using metal ions and nanoparticles: From mechanisms to applications. *Bioact Mater.* **6** (12), 4470–4490 (2021). 34027235 10.1016/j.bioactmat.2021.04.033 PMC8131399

[67] Khatoon, U. T., Velidandi, A. & Nageswara Rao, G. V. S. Copper oxide nanoparticles: Synthesis via chemical reduction, characterization, antibacterial activity, and possible mechanism involved. *Inorg. Chem. Commun.* **149**, 110372 (2023).

[68] De Simone, S. et al. Development of silver nano-coatings on silk sutures as a novel approach against surgical infections. *J. Mater. Sci. Mater. Med.* **25** (9), 2205–2214 (2014). 24997984 10.1007/s10856-014-5262-9

[69] Mongy, Y. & Shalaby, T. Green synthesis of zinc oxide nanoparticles using Rhus coriaria extract and their anticancer activity against triple-negative breast cancer cells. *Sci. Rep.* **14** (1), 13470 (2024). 10.1038/s41598-024-63258-7 PMC11169510 38866790

