# Supplementary figures and images for: Preparation and characterization of antibacterial fibrous membranes composites based on green synthesized nanoparticles loaded on electrospun polyacrylonitrile fibrous membranes
Source: Sci Rep. 2026 May 19;16:15397. doi: 10.1038/s41598-026-51833-z (PMC13184077; doi:10.1038/s41598-026-51833-z)

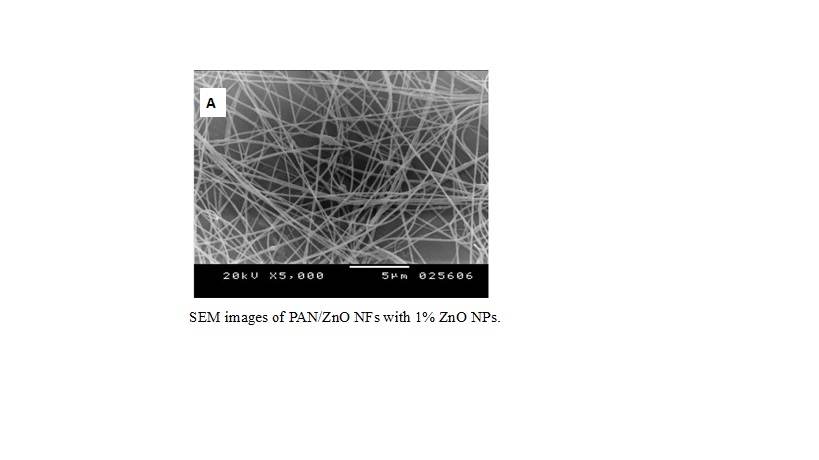

Supplement: Supplementary file 1 — Supplementary Material 1 [file 41598_2026_51833_MOESM1_ESM.jpg]

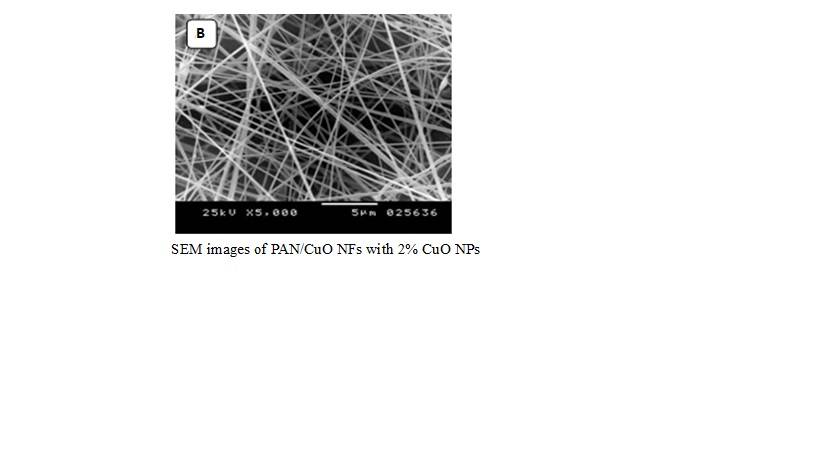

Supplement: Supplementary file 2 — Supplementary Material 2 [file 41598_2026_51833_MOESM2_ESM.jpg]

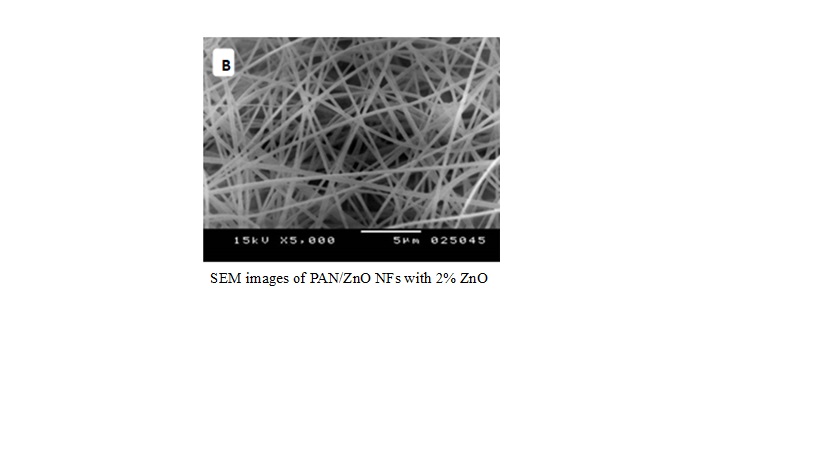

Supplement: Supplementary file 3 — Supplementary Material 3 [file 41598_2026_51833_MOESM3_ESM.jpg]

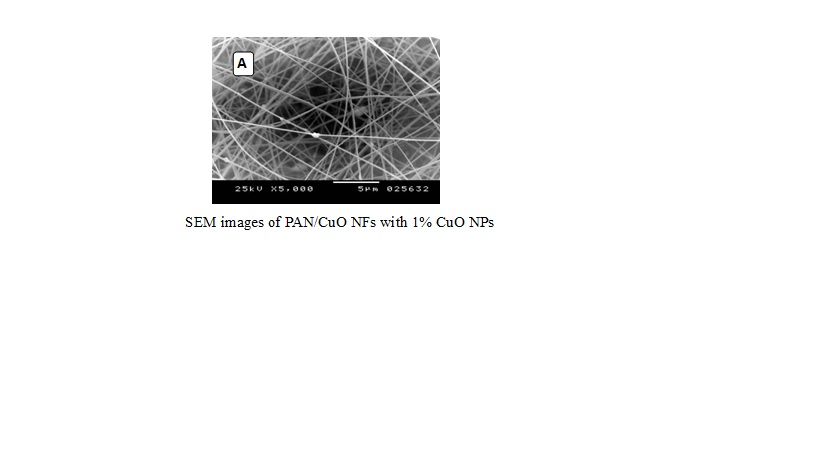

Supplement: Supplementary file 4 — Supplementary Material 4 [file 41598_2026_51833_MOESM4_ESM.jpg]
